# A multi-analytical characterization of fourteenth to eighteenth century pottery from the Kongo kingdom, Central Africa

**DOI:** 10.1038/s41598-022-14089-x

**Published:** 2022-06-15

**Authors:** Anna Tsoupra, Bernard Clist, Maria da Conceição Lopes, Patricia Moita, Pedro Barrulas, Maria da Piedade de Jesus, Sónia da Silva Domingos, Koen Bostoen, José Mirao

**Affiliations:** 1 HERCULES Laboratory, University of Évora, Palácio do Vimioso, Largo Marquês de Marialva 8, 7000-809 Évora, Portugal; 2 Institut des Mondes Africains (IMAF), Paris, France; 3 Research Center in Archaeology, Arts and Heritage Sciences, University of Coimbra, Coimbra, Portugal; 4 Ministério da Cultura, Rua do MAT Complexo Administrativo, “Clássico do Talatona”, Luanda, Angola; 5 Centro Nacional de Investigação Científica (CNIC), Luanda, Angola; 6 Department of Languages and Cultures, BantUGent – UGent Centre for Bantu Studies, Ghent University, Ghent, Belgium; 7 Department of Geosciences, School of Science and Technology, University of Évora, Colégio Luís António Verney, Évora, Portugal

**Keywords:** Socioeconomic scenarios, Geochemistry, Mineralogy

## Abstract

Pottery traditions reflect the socioeconomic framework of past cultures, while the spatial distribution of pottery indicates exchange patterns and interaction processes. Material and earth sciences are employed here to determine raw material sourcing, selection and processing. The Kongo kingdom, internationally renowned since the late fifteenth century, is one of the most famous precolonial states in Central Africa. Despite the large number of historical studies relying on African and European oral and written chronicles, there are still considerable gaps in our current understanding of this political unit. Here, we provide new insights into pottery production and circulation within the Kongo kingdom. Implementing a multi-analytical approach, namely XRD, TGA, petrographic analysis, XRF, VP-SEM-EDS and ICP-MS, on selected samples, we determined their petrographic, mineralogical and geochemical signatures. Our results allowed us to correlate the archaeological objects to naturally occurring materials and to establish ceramic traditions. We identified production templates, exchange patterns, distribution of high-quality goods and interaction processes through technological knowledge transmission. Our results demonstrate that political centralisation in the Lower Congo region of Central Africa had a direct impact on pottery production and circulation. We expect our study to provide a sound basis for further comparative research to contextualise the region.

## Introduction

Pottery making and use have been central activities in many cultures and their socio-political context had a great impact on the production organisation and manufacturing process of these objects^1,2^. In this framework, ceramic studies can enhance our knowledge regarding past societies^3,4^. By examining archaeological ceramics, we can correlate their attributes to specific ceramic traditions and subsequently to production patterns^1,4,5^. As noted by Matson^6^, based on *ceramic ecology*, the selection of raw materials is related to the spatial availability of natural sources. Moreover, considering various ethnographic case studies, Whitbread^2^ refers to an 84% probability of source exploitation being within a 7 km radius from the ceramic production place, where in Africa, a 3 km radius with an 80% probability has been suggested^7^. Nevertheless, it is important not to neglect the dependence of the production organisation on technological factors^2,3^. Technological choices can be studied by investigating the interrelations between materials, techniques and technological knowledge^3,8,9^. A sequence of such choices can define a specific ceramic tradition. At this point, the integration of archaeometry into the research contributes significantly^3,10–12^ to a better understanding of past societies. The application of a multi-analytical approach can address questions regarding all the phases involved in a *chaîne opératoire*, such as natural resource exploitation and raw materials selection, procurement and processing^3,10–12^.

This study focuses on the Kongo kingdom, one of the most influential polities that developed in Central Africa. Before the emergence of modern states, Central Africa was made up of a complex socio-political mosaic, characterised by great cultural and political variability, with structures that ranged from small and decentralised to complex and highly centralized political domains^13–15^. Within this socio-political context, the Kongo kingdom is assumed to have emerged in the fourteenth century out of an aggregation of three bordering federations^16,17^. In its heydays, it covered an area roughly corresponding to the area between the Atlantic Ocean to the west and the Kwango River to the east in the current-day Democratic Republic of Congo (DRC) and the northern part of present-day Angola down to the latitude of Luanda. It played a key role in the broader region during its apogee and underwent a development towards more complexity and centralisation until the eighteenth century^14,18–21^. Social stratification, common currency, taxation system, specific labour distribution and slave trade^18,19^ reflect a model of political economy, as defined by Earle^22^. From its foundation until the late seventeenth century, the Kongo kingdom expanded significantly and established strong ties with Europe from 1483 onwards, through which it also participated in the Atlantic trade^18–20,23–25^ (see more detailed historical information in Supplement 1).

**Figure 1:**
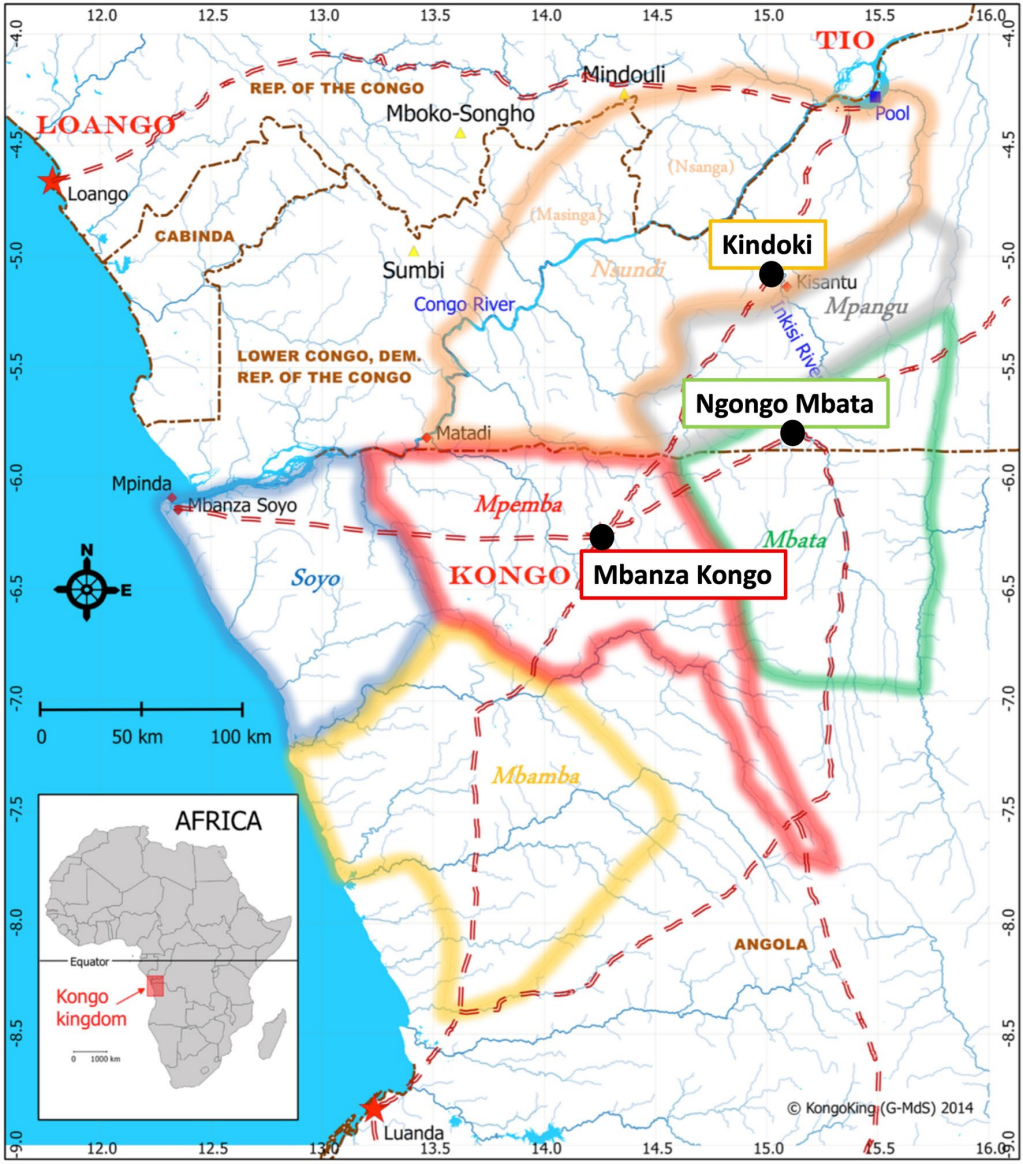
The Kongo kingdom and its six main provinces (Mpemba, Nsundi, Mbata, Soyo, Mbamba, Mpangu) in the Sixteenth to seventeenth centuries. The three sites (Mbanza Kongo, Kindoki and Ngongo Mbata) discussed in this study are shown on the map.

Approaches from material and earth sciences have been applied to ceramic artefacts from three archaeological sites of the Kongo kingdom, where excavation campaigns were carried out in the past decade, i.e. Mbanza Kongo in Angola and Kindoki and Ngongo Mbata in the DRC (Fig. 1) (see archaeological data in Supplement 2). Mbanza Kongo, recently added to the UNESCO World Heritage List, was located in the Mpemba province of the ancient polity. Situated on a central plateau at the intersection of the most important trade routes, it was the kingdom’s political and administrative capital hosting the king’s throne^21,26,27^. Kindoki and Ngongo Mbata were located within the Nsundi and Mbata provinces, respectively, and before the kingdom’s formation, these provinces may have been part of the Seven Kingdoms of Kongo dia Nlaza—one of the incorporated polities^28,29^. Both of them played an important role in the kingdom’s entire history^17^. The archaeological sites of Kindoki and Ngongo Mbata are located in the Inkisi Valley in the northern part of the kingdom, one of the first regions that the kingdom’s founding father would have conquered. The provincial capital Mbanza Nsundi hosting the Kindoki site was traditionally ruled by later successors of Kongo kings^17,18,30^. The province of Mbata was situated mostly east of the Inkisi River^31^. The rulers of Mbata (and to a certain extent Soyo) had the historical privilege to be the only ones to be selected from the local nobility through inheritance, as opposed to other provinces the kingdom whose rulers were appointed by the royal court itself, which implied greater mobility^18,26^. Ngongo Mbata, although not the provincial capital of Mbata, played a central role during at least the seventeenth century. Due to its strategic location within an exchange network, Ngongo Mbata contributed to the province’s development as an important trade market^16–18,26,31,32^.

Until a decade ago, archaeological knowledge of the Kongo kingdom was limited^33^. Most insights into the kingdom’s history were based on local oral traditions and on African and European written sources^16,17^. Due to the absence of systematic archaeological research, the chrono-cultural sequence of the Kongo area was fragmented and incomplete^34^. Archaeological excavation campaigns since 2011 have aimed to fill these gaps and have led to the discovery of important structures, features and artefacts. Among the findings, potshards are unmistakably the most important find^29–32,35,36^. Regarding Central Africa’s Iron Age, archaeometric projects such as the present one are extremely rare^37,38^.

**Figure 2:**
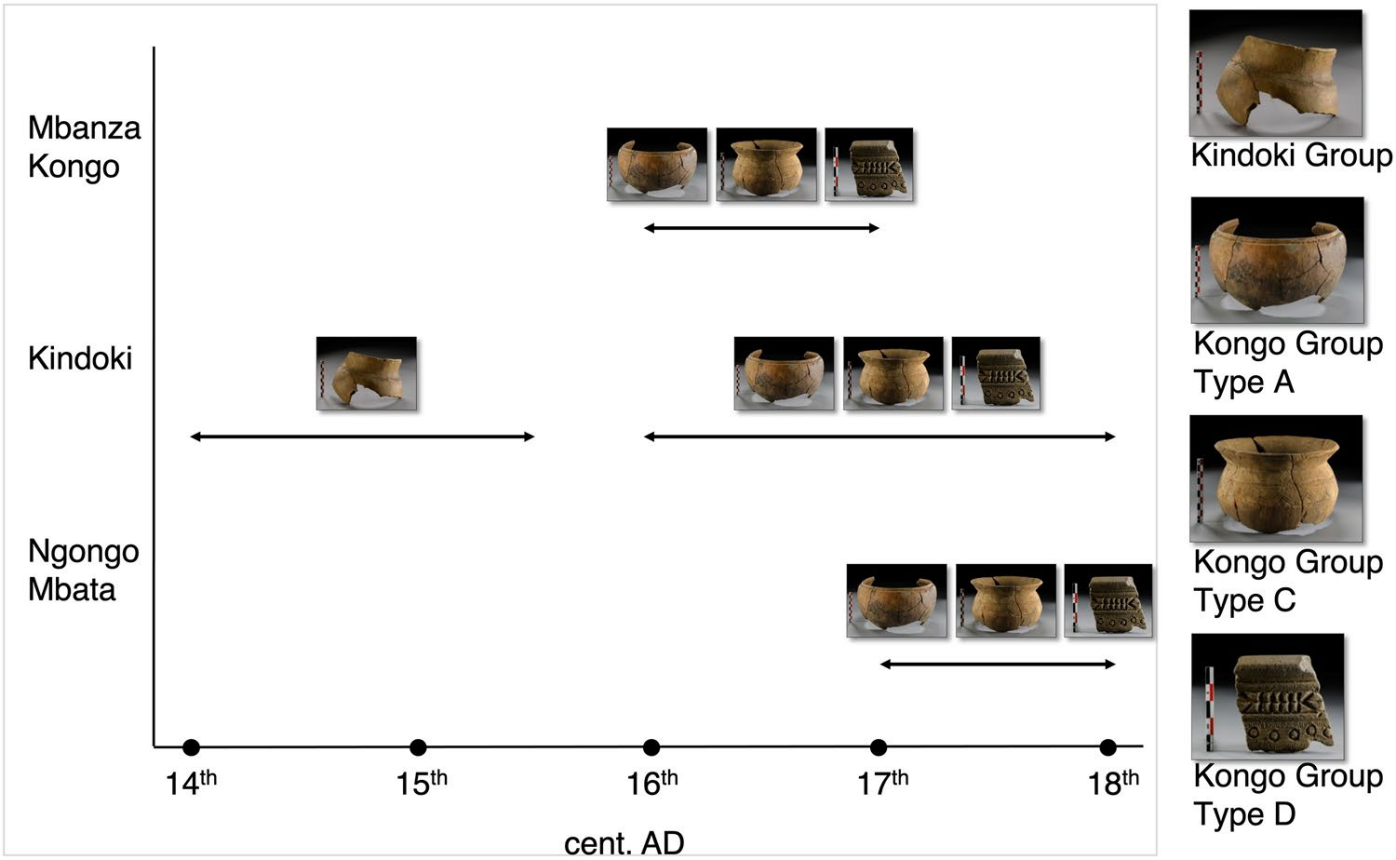
Illustration of the four typological groups (Kindoki Group and Kongo Group: Types A, C and D) of pottery from Kongo Kingdom discussed in this study; graphic representation of their chronological occurrence at each archaeological site, Mbanza Kongo, Kindoki and Ngongo Mbata.

We present the results of the mineralogical, geochemical and petrographic analyses conducted on a group of potshards from the three excavated Kongo kingdom areas (see archaeological data in Supplement 2). The samples belong to four pottery types (Fig. 2), one type from the Kindoki Group and three from the Kongo Group^30,31,35^. The Kindoki Group dates back to the early kingdom period (fourteenth–mid fifteenth century). Among the sites discussed in this study, Kindoki (n = 31) is the only one where the Kindoki Group is attested^30,35^. The three types from the Kongo Group—Types A, C and D—date back to the later kingdom period (sixteenth–eighteenth century) and are concurrently present in the three archaeological sites considered here^30,31,35^. Kongo Type C pots are cooking pots, which are abundant at all three sites^35^. Kongo Type A pots were presumably used as serving pots and are represented by only a small number of shards^30,31,35^. Kongo Type D ceramics are supposed to have had a domestic use only—as they have never found so far as a funerary deposit in tombs—and have been associated with a specific elite group of users^30,31,35^. Their shards also occur in small quantities only. The Type A and Type D pots show a similar spatial distribution in the Kindoki and Ngongo Mbata sites^30,31^. In Ngongo Mbata, 37,013 potshards of Kongo Type C dominated the assemblage by far, with only 193 shards of Kongo Type A and 168 of Kongo Type D^31^.

X-ray diffraction (XRD), thermogravimetric analysis (TGA), petrographic analysis, variable pressure scanning electron microscopy coupled to energy dispersive X-ray spectroscopy (VP-SEM-EDS), X-ray fluorescence spectroscopy (XRF) and inductively coupled plasma mass spectrometry (ICP-MS) have been implemented to address issues regarding potential sources of raw materials and production technology. We aim at identifying ceramic traditions and by linking them to certain production patterns to shed new light on the social structure of one of Central Africa’s most illustrious political entities.

## Geological framework

**Figure 3:**
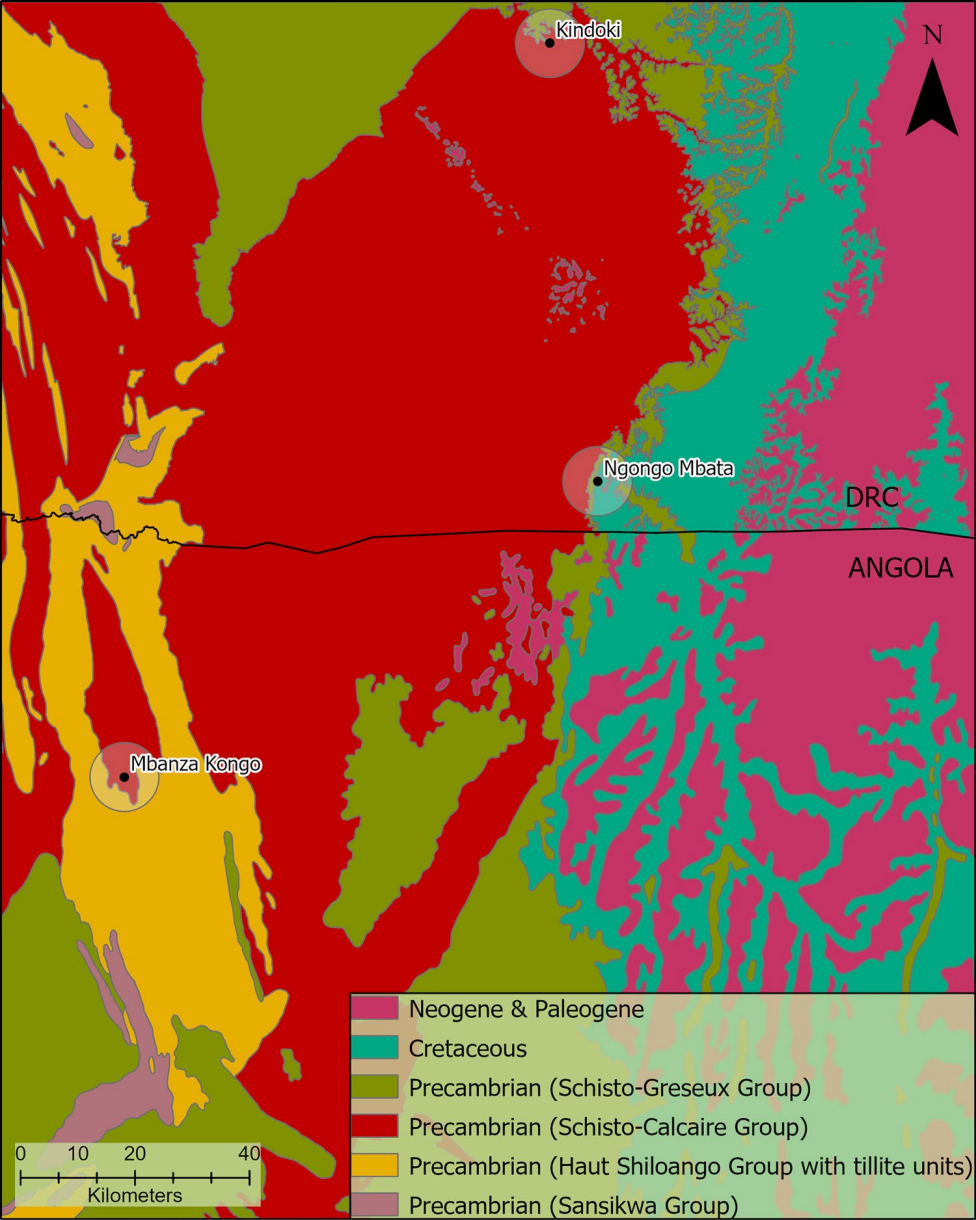
Geological map of the studied region. The three archaeological sites (Mbanza Kongo, Kindoki and Ngongo Mbata) are displayed on the map. The circle around the sites indicates a 7 km radius, which corresponds to an 84% probability of source exploitation^2^. The map refers to DRC and Angola with the border line demonstrated on it. The geological map (shapefile in Supplement 11) was created in ArcGIS Pro 2.9.1 software (URL: https://www.arcgis.com/), with reference to Angolan^41^ and Congolese^42,65^ geological maps (raster files), produced using different cartographic criteria.

The Kongo kingdom case is particularly challenging for provenance studies due to the diversity and peculiarity displayed in the local geology (Fig. 3). The regional geology is discernible by the presence of a slightly to non-deformed geological sedimentary and metamorphic sequence, known as the *West Congolian* Supergroup. In a bottom-up approach, the sequence begins with rhythmical alternating quartzite-claystone formations from the *Sansikwa* Group, followed by the *Haut Shiloango* Group, which is characterized by the presence of stromatolitic carbonates, and in the DRC, tillite units were identified close to the bottom and the top of the group. The Neoproterozoic *Schisto-Calcaire* Group is a carbonate-pelite assemblage with some Cu–Pb–Zn mineralizations. This geological formation manifests an unusual process through the weak diagenesis of Mg-clay^39^ or the slight alteration of dolostone producing talc^40^. This results in the simultaneous presence of calcareous and talc mineral sources. This unit is overlain by the Precambrian *Schisto-Greseux* Group composed of sand-pelitic red beds.

Above a sedimentary discontinuity, the *Cretaceous* units are composed of continental sedimentary rocks, such as sandstones and claystones. In the vicinity, this geological formation is known as a secondary sedimentary source of diamonds, after the erosion of *early-Cretaceous* kimberlite pipes^41,42^. Further igneous and high-grade metamorphic rocks are not reported in this region.

The region surrounding Mbanza Kongo is characterized by the presence of clastic and chemical sediments on Precambrian formations, mainly limestone and dolostone of the *Schisto-Calcaire* Group and slate, quartzite and graywacke of the *Haut Shiloango* Group^41^. The closest geological units to the Kindoki archaeological site are Holocene alluvium sedimentary rocks and limestone, slates and cherts of the *Schisto-Calcaire* Group, overlain by the feldspar-quartzites of the Precambrian *Schisto-Greseux* Group. Ngongo Mbata is in a narrow band of *Schisto-Greseux* rocks between the older *Schisto-Calcaire* Group and the nearby *Cretaceous* red sandstones^42^. Moreover, a kimberlite source, known as Kimpangu, is reported in an off-craton setting in the Lower Congo region, in the wider vicinity of Ngongo Mbata^43^.

## Results

### Ceramic mineralogical composition

**Figure 4:**
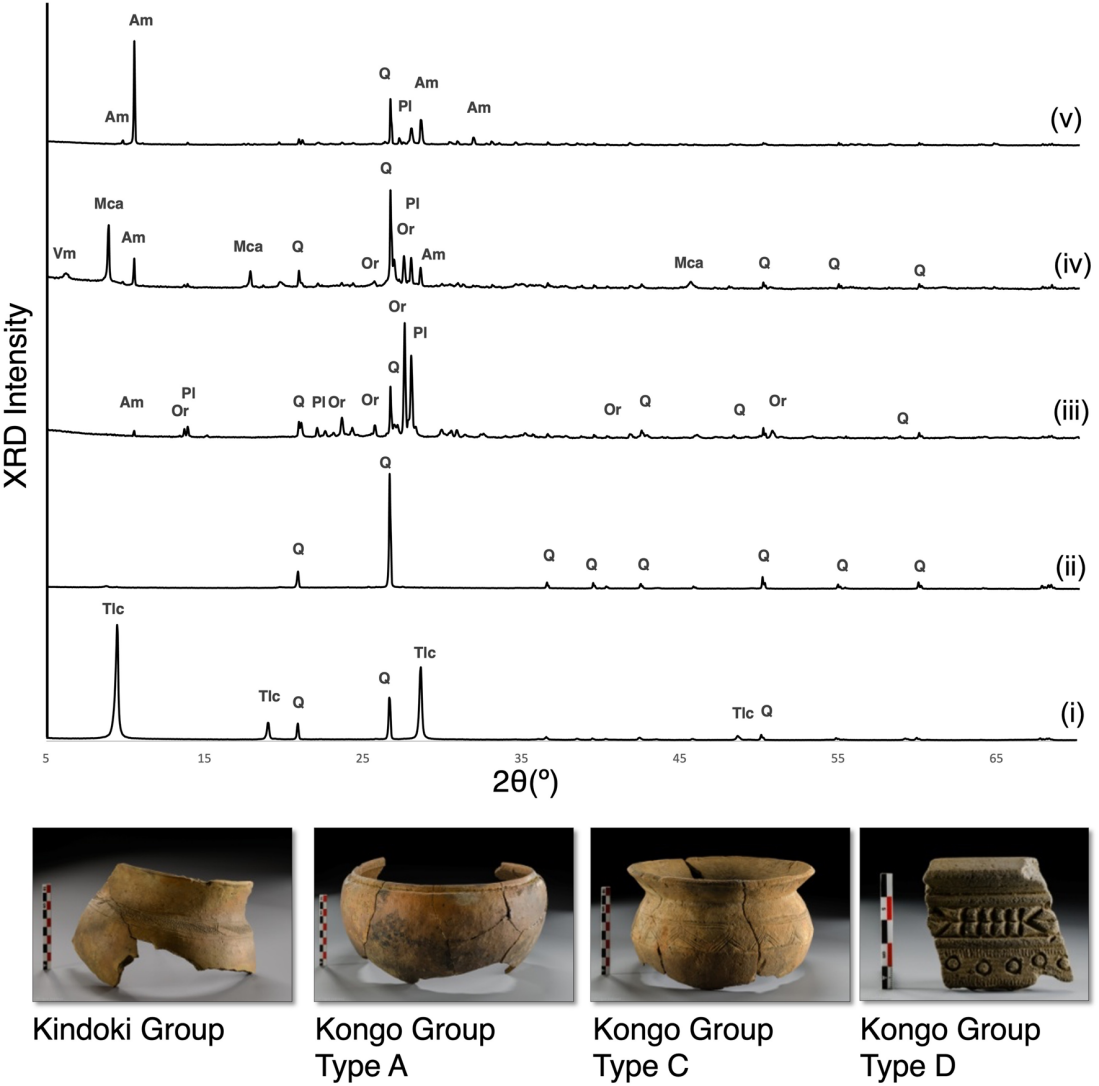
Representative XRD patterns from Kongo kingdom pottery, on the basis of the main crystalline phase, corresponding with the typological groups: (i) talc-rich composition encountered in samples from Kindoki Group and Kongo Type C, (ii) quartz-rich composition encountered in samples from Kindoki Group and Kongo Type C, (iii) feldspar-rich composition encountered in samples from Kongo Type A and Kongo Type D, (iv) mica-rich composition encountered in samples from Kongo Type A and Kongo Type D, (v) amphibole-rich composition encountered in samples from Kongo Type A and Kongo Type D. *Q* quartz, *Pl* plagioclase, *Or* potassium feldspar, *Am* amphibole, *Mca* mica, *Tlc* talc, *Vrm* vermiculite.

The semi-quantitative results of the major mineral phases obtained by XRD are presented in Table 1, and representative XRD patterns are shown in Fig. 4. Quartz (SiO_2_) is the main mineral phase, regularly associated with potassium feldspars (KAlSi_3_O_8_), micas [e.g., KAl_2_(Si_3_Al)O_12_(OH)_2_], and/or talc [Mg_3_Si_4_O_10_(OH)_2_]. Plagioclase minerals [XAl_(1–2)_Si_(3–2)_O_8_, X = Na or Ca] (i.e. sodium and/or calcium feldspars) and amphiboles [(X)_(0–3)_[(Z)_(5–7)_(Si, Al)_8_O_22_(O,OH,F)_2_, X = Ca^2+^, Na^+^, K^+^, Z = Mg^2+^, Fe^2+^, Fe^3+^, Mn^2+^, Al, Ti] are interrelated crystalline phases, commonly with micas. Amphiboles are not usually present with talc.

**Table 1:** RIR-XRD results of the major mineral phases of the samples from Mbanza Kongo (MBK), Kindoki (KDK) and Ngongo Mbata (NBC).

Sample no.	Type	Q%	Pl%	Or%	Am%	Mca%	Tlc%	Vrm%
MBK_S.2	A	43.0	8.4	19.8	5.9	22.8		
MBK_S.6	A	13.2	39.1	4.4	19.5	23.2		0.6
MBK_S.11	A	49.2	4.9	15.6	4.0	26.3		
MBK_S.20	A	12.3	5.3	22.0	17.5	42.3		0.1
MBK_S.1	C	55.4		12.1		31.5		
MBK_S.3	C	6.8					90.7	
MBK_S.8	C	63.2	22.0	11.3	3.5			
MBK_S.9	C	24.8	22.5	4.2	17.3	30.9		0.4
MBK_S.10	C	51.0		14.1		30.0	4.1	
MBK_S.12	C	8.0		3.1		1.3	87.6	
MBK_S.14	C	11.1					88.9	
MBK_S.17	C	44.6	1.0	40.6		13.9		
MBK_S.21	C	93.5	0.9	3.2		2.4		
MBK_S.23	C	69.0	10.1	9.5	3.1	8.3		
MBK_S.4	D	13.3	37.3	35.6	13.7			
MBK_S.5	D	21.6	43.5	27.3	5.5	2.1		
MBK_S.7	D	34.5	10.5	19.0	13.9	22.0		
MBK_S.15	D	19.3	5.6	8.8	59.9		6.2	0.3
MBK_S.16	D	69.9	13.7	11.0	5.4			
MBK_S.19	D	18.4	6.3	5.2	68.7		1.3	
MBK_S.22	D	35.6	44.9	15.4		4.1		
MBK_S.24	D	15.5	23.9	52.6	4.3	3.3	0.4	
MBK_S.25	D	32.2	36.7	21.8	2.7	6.7		
KDK_S.11	KDK	48.6		7.2		34.7	9.2	0.3
KDK_S.12	KDK	37.5		6.7	2.4	45.5	6.3	0.9
KDK_S.13	KDK	32.7		6.5		23.8	35.4	0.8
KDK_S.14	KDK	65.2	1.8	6.5		25.6		
KDK_S.15	KDK	43.2		7.3		30.2	18.5	0.2
KDK_S.16	KDK	31.1		2.7		24.5	39.3	2.5
KDK_S.6	A	11.7	22.6	21.5	22.6	21.5		
KDK_S.7	A	7.0	9.1	10.1	44.9	28.2		0.8
KDK_S.8	A	6.5	35.4	17.8	17.1	22.9		0.4
KDK_S.9	A	14.9	19.3	5.6	22.3	37.9		
KDK_S.17	C	66.9				17.9	14.5	
KDK_S.18	C	91.5		7.9				
KDK_S.19	C	1.6	4.4	7.0	24.5	61.3		1.2
KDK_S.20	C	4.3					95.7	
KDK_S.21	C	89.0				11.0		
KDK_S.22	C	97.4		2.5				
KDK_S.23	C	95.5				3.8		
KDK_S.25	C	10.7	8.7	11.5	22.0	46.2		0.8
KDK_S.1	D	14.1	19.4	54.9	10.6	1.0		
KDK_S.2	D	24.7	42.3	26.4		6.7		
KDK_S.3	D	20.3	25.0	41.7	10.8	2.3		
KDK_S.4	D	11.5	44.1	41.0	3.4			
KDK_S.5	D	11.7	55.3	27.4	5.7			
NBC_S.1	A	7.5	11.6	8.0	40.6	30.8		0.6
NBC_S.2	A	10.8	14.7	13.9	11.2	49.2		0.1
NBC_S.3	A	52.8	16.7		4.0		26.6	
NBC_S.4	A	9.4	3.9	1.3	85.5			
NBC_S.5	A	12.1	4.8	10.4	17.5	55.0		0.3
NBC_S.9	C	92.6		4.2		2.9		0.3
NBC_S.10	C	92.2		1.6		5.7	0.5	
NBC_S.11	C	91.4		1.5		4.7	2.4	
NBC_S.12	C	96.9		1.6			1.5	
NBC_S.13	C	90.4		5.2		4.4		
NBC_S.14	C	86.2		3.8		10.0		
NBC_S.16	D	12.9	25.3	56.1	5.7			
NBC_S.17	D	38.8	21.3	29.5	7.5	2.9		
NBC_S.18	D	30.7	27.4	37.9	4.0			
NBC_S.19	D	20.9	33.6	41.4	4.2			
NBC_S.20	D	14.4	60.1	13.7	11.9			
NBC_S.21	D	20.8	16.8	14.8	35.8	11.9		
NBC_S.22	D	19.7	26.3	23.0	20.4	10.7		
NBC_S.23	D	6.2	3.9	1.1	88.9			
NBC_S.24	D	20.2	21.6	12.8	2.4	42.9		
NBC_S.25	D	29.1	13.2	22.3	27.3	8.0		

*Q* quartz, *Pl* plagioclase, *Or* potassium feldspar, *Am* amphibole, *Mca* mica, *Tlc* talc, *Vrm* vermiculite.

The undistinguishable XRD profiles of talc, Mg_3_Si_4_O_10_(OH)_2_ and pyrophyllite, Al_2_Si_4_O_10_(OH)_2_, required a complementary technique to identify their presence, absence or possible coexistence. TGA was performed on three representative samples (MBK_S.14, KDK_S.13 and KDK_S.20). The TG curves (Supplement 3) are in accordance with the presence of the talc mineral phase and the absence of pyrophyllite. The dehydroxylation and structure decomposition, observed between 850 and 1000 °C, corresponds to talc. No mass loss is observed between 650 and 850 °C, indicating the absence of pyrophyllite^44^.

As a minor phase, vermiculite [(Mg, Fe^+2^, Fe^+3^)_3_[(Al, Si)_4_O_10_](OH)_2_·4H_2_O], identified by the analysis of oriented aggregate mounts of representative samples with a peak at 16–7 Å, is detected mainly in the samples of the Kindoki Group and in those of the Kongo Group of Type A.

The samples from the Kindoki Group type recovered from the wider area around Kindoki present a mineralogical composition that is marked by the presence of talc, the abundance of quartz and micas and the presence of potassium feldspars.

The mineralogical composition of the Kongo Type A samples is characterized by the abundance of quartz-mica pair with different ratios and the presence of potassium feldspars, plagioclases, amphiboles and micas. The abundance of amphiboles and feldspars marks this typological group, especially in the Kongo Type A samples from Kindoki and Ngongo Mbata.

The samples of Kongo Type C present a diverse mineralogical composition within the typological group, which is highly dependent on the archaeological site. The samples from Ngongo Mbata are strongly enriched in quartz and present a consistent composition. Quartz is also a dominant phase in the Kongo Type C samples from Mbanza Kongo and Kindoki, but in these cases, some samples are enriched in talc and micas.

Kongo Type D has a distinct mineralogical composition at all three archaeological sites. In this pottery type, feldspars, especially plagioclases, are very abundant. Amphiboles are usually present in significant amounts. Quartz and micas are represented. The relative amounts vary between different samples. Talc was detected in the amphibole-rich shards of this typological group in Mbanza Kongo.

### Petrofabric and temper mineral composition

The main tempering minerals identified by petrographic analysis are quartz, feldspars, micas and amphiboles. The rock inclusions consist of medium- and high-grade metamorphic, igneous and sedimentary rock fragments. The fabric data, obtained using reference charts by Orton^45^, show a temper sorting ranging from poorly to well sorted with a ratio of temper-matrix from 5 to 50%. The temper grains are from rounded to angular without a preferential orientation.

**Figure 5:**
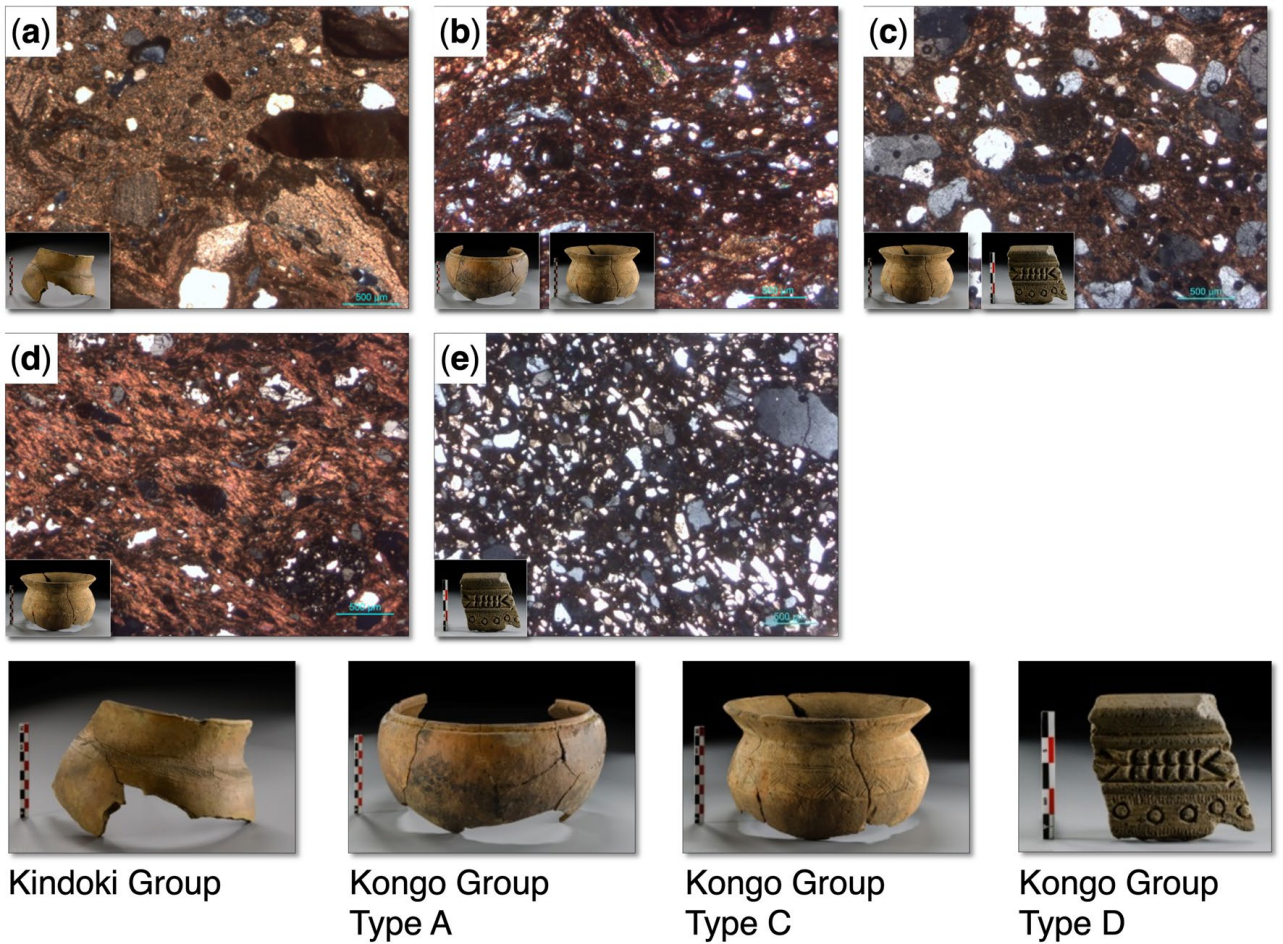
Representative optical microphotographs of the examined thin-section from the Kongo Kingdom pottery; correspondence of the petrographic with the typological groups. (**a**) PGa group, (**b**) PGb group, (**c**) PGc group, (**d**) PGd group and (**e**) PGe group.

Based on the textural and mineralogical variations, five petrographic groups (PGa, PGb, PGc, PGd and PGe) were distinguished. PGa group: low ratio temper-matrix (5–10%), fine texture of the matrix and presence of big sedimentary and metamorphic rock inclusions (a in Fig. 5); PGb group: relatively high ratio temper-matrix (20–30%), poor temper sorting, angular temper grains and high presence of phyllosilicates, micas and big rock inclusions of medium and high-grade metamorphic rocks (b in Fig. 5); PGc group: relatively high ratio temper-matrix (20–40%), well to very well temper sorting, small to very small rounded temper grains, abundance of quartz grains and occasionally presence planar voids (c in Fig. 5); PGd group: low ratio temper-matrix (5–20%), small temper grains, big rock inclusions poorly sorted, fine texture of the matrix (d in Fig. 5); and PGe group: high ratio temper-matrix (40–50%), well to very well temper sorting, two-sizes of temper grains and diverse mineralogical composition in terms of temper (e in Fig. 5). Figure 5 displays representative optical microphotographs of the petrographic groups. The optical investigation of the samples resulted in strong correlations between the typological classification and the petrographic groups, especially in the samples from Kindoki and Ngongo Mbata (see representative microphotographs from the whole sample-set in Supplement 4).

The Kindoki Group samples comprise a well-defined petrographic group correlated with the PGa petrographic group. The Kongo Type A samples are highly associated with the PGb petrographic group, besides Kongo Type A sample NBC_S.4 Kongo Type A from Ngongo Mbata, which in terms of sorting is related to the PGe group. Most of the Kongo Type C samples from Kindoki and Ngongo Mbata, as well as the Kongo Type C samples MBK_S.21 and MBK_S.23, from Mbanza Kongo belong to the PGc group. However, several Kongo Type C samples show characteristics of other petrographic groups. The Kongo Type C samples MBK_S.17 and NBC_S.13 present textural attributes correlated with the PGe group. The Kongo Type C samples MBK_S.3, MBK_S.12 and MBK_S.14, comprise a separate petrographic group, PGd, while the Kongo Type C samples KDK_S.19, KDK_S.20 and KDK_S.25 share similar attributes with the PGb group. The Kongo Type C sample MBK_S.14 could be considered an outlier due to its porfiroclastic texture. Almost all the samples belonging to Kongo Type D are correlated with the PGe petrographic group, except the Kongo Type D samples MBK_S.7 and MBK_S.15 from Mbanza Kongo, which present larger temper grains at a lower density (30%) closer to the PGc group.

**Figure 6:**
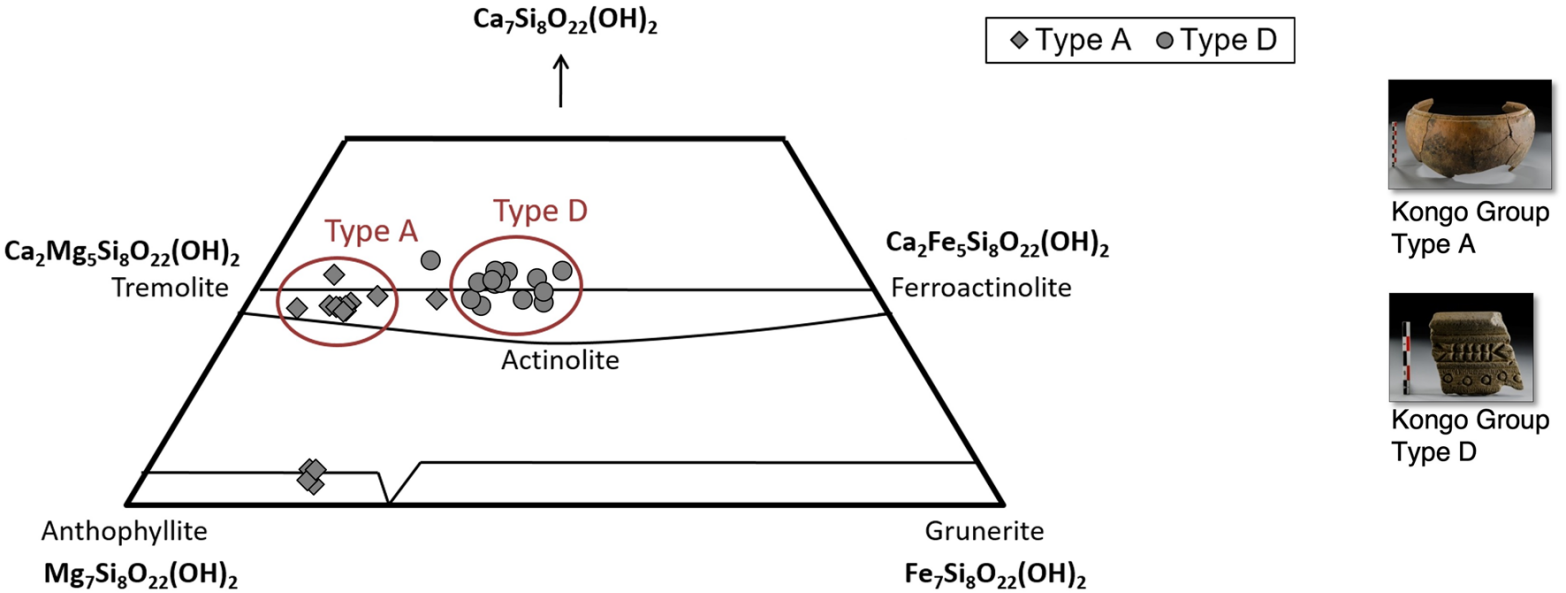
VP-SEM-EDS data. Ternary diagram illustrating the different compositions of amphiboles between Kongo Type A and Kongo Type D pots on selected samples from Mbanza Kongo (MBK), Kindoki (KDK) and Ngongo Mbata (NBC); symbol coded by typological group.

Samples from the three archaeological sites were analyzed by VP-SEM-EDS to illustrate the elemental distribution and to determine the major elemental composition of individual temper grains. The EDS data allowed the identification of quartz, feldspars, amphiboles, iron oxides (hematite), titanium oxides (e.g., rutile), titanium-iron oxides (ilmenite), zirconium silicate (zircon) and calcium-titanium nesosilicates (sphene). Silica, aluminium, potassium, calcium, sodium, titanium, iron and magnesium are the most common chemical elements of the matrix. The consistently higher magnesium concentrations in the Kindoki Group and the Kongo Type A pots can be explained by the presence of talc or Mg-clay minerals. Based on the elemental analysis, the feldspar grains correspond mainly to potassium feldspars, albite, oligoclase and occasionally to labradorite and anorthite (Supplement 5, Figs. S8–S10), while the amphibole grains are tremolite, actinolite and in the case of the Kongo Type A sample NBC_S.3, anthophyllite. A clear differentiation is observed in the composition of the amphiboles (Fig. 6) in Kongo Type A (tremolite) and Kongo Type D ceramics (actinolite). Moreover, at the three archaeological sites, ilmenite grains are strongly related to the Type D samples. A high manganese content is identified in the ilmenite grains. Nevertheless, this does not change their common iron–titanium (Fe–Ti) substitution mechanism^46^ (see Supplement 5, Fig. S11).

In accordance with the XRD results, quartz and potassium feldspars are the major minerals in the Kongo Type C samples, while the presence of quartz, potassium feldspars, albite, anorthite and tremolite characterizes the Kongo Type A samples. The Kongo Type D samples show quartz, potassium feldspars, albite, oligoclase, Mn-ilmenites and actinolite as the main mineral components. The Kongo Type A sample NBC_S.3 could be considered an outlier, since its plagioclase is labradorite and the amphibole is anthophyllite and the presence of Mn-ilmenites was registered. The Kongo Type C sample NBC_S.14 also contains Mn-ilmenite grains (Supplement 5, Figs. S12–S15).

### Ceramic chemical composition

XRF analysis of representative samples from the three archaeological sites was performed to identify major element groups. The major element compositions are presented in Table 2. The analysed samples show enrichment in silicon and aluminium oxides and present a calcium oxide concentration lower than 6%. The high concentration of magnesium is attributed to the presence of talc, and it shows an inverse correlation with silicon and aluminium oxides. The higher sodium and calcium oxide contents are consistent with the abundance of plagioclases.

**Table 2:** Major elemental compositions (wt%) obtained by the XRF analysis of the samples from Mbanza Kongo (MBK), Kindoki (KDK) and Ngongo Mbata (NBC).

Sample no.	Type	Na_2_O	MgO	Al_2_O_3_	SiO_2_	P_2_O_5_	K_2_O	CaO	TiO_2_	MnO	Fe_2_O_3_
KDK_S.12	KDK	0.18	8.40	15.90	53.20	0.58	2.50	0.37	0.82	0.03	3.98
KDK_S.13	KDK	0.14	9.81	15.60	49.70	0.98	1.69	0.16	0.61	< 0.01	3.90
KDK_S.15	KDK	0.15	9.61	12.60	61.00	0.17	1.90	0.07	0.61	< 0.01	1.91
MBK_S.6	A	0.78	3.71	16.70	49.90	0.08	3.44	2.47	0.84	0.08	8.50
MBK_S.11	A	0.44	1.64	16.20	50.40	1.86	3.80	1.23	0.91	0.05	7.79
KDK_S.6	A	1.40	2.98	16.90	51.60	0.04	4.44	3.06	0.78	0.06	8.79
KDK_S.8	A	0.80	2.92	17.50	49.40	0.04	3.74	3.29	0.75	0.17	8.63
NBC_S.3	A	0.37	5.16	17.10	53.90	0.24	0.14	1.57	0.65	1.25	7.57
NBC_S.4	A	1.20	4.59	15.60	48.50	0.60	0.15	6.09	0.62	0.16	12.00
NBC_S.5	A	0.78	3.22	17.40	49.50	0.78	3.73	2.07	0.84	0.10	8.44
MBK_S.9	C	1.69	2.85	16.80	46.10	1.35	1.18	4.68	1.50	0.07	10.40
MBK_S.14	C	0.13	13.90	7.12	66.90	0.29	0.43	0.31	0.19	0.01	2.12
MBK_S.17	C	0.35	0.35	18.70	60.20	0.80	2.84	0.18	0.83	n.d.	7.68
MBK_S.21	C	0.21	0.64	15.30	67.10	0.89	0.99	0.57	0.68	0.04	5.16
KDK_S.17	C	0.44	2.87	15.10	61.80	0.08	0.73	0.19	0.85	0.01	5.01
KDK_S.19	C	0.66	5.03	16.70	49.20	0.07	3.90	3.20	0.74	0.02	9.81
KDK_S.20	C	0.19	20.70	5.55	58.00	0.64	0.33	0.21	0.19	n.d.	2.13
NBC_S.11	C	0.37	0.61	16.40	61.70	1.74	0.54	0.27	0.53	0.01	2.61
NBC_S.12	C	0.20	0.70	16.30	70.10	0.43	0.41	0.17	0.54	0.01	3.22
NBC_S.14	C	0.21	0.53	14.90	62.50	1.74	0.25	0.29	0.78	0.02	3.06
MBK_S.4	D	3.01	0.44	16.80	55.80	0.20	4.10	1.42	3.35	0.20	6.36
MBK_S.15	D	1.64	2.92	16.50	51.60	0.78	1.29	4.14	1.17	0.17	10.00
MBK_S.19	D	1.14	7.55	9.63	52.80	1.00	0.86	6.09	0.73	0.21	10.30
MBK_S.22	D	2.20	0.52	15.40	62.90	0.39	2.80	2.21	0.78	0.05	5.14
MBK_S.25	D	1.99	0.98	17.70	54.70	0.75	2.78	1.65	0.90	0.19	7.53
KDK_S.1	D	2.65	0.37	17.40	54.60	0.15	4.17	1.10	2.63	0.15	6.40
KDK_S.4	D	4.28	0.47	18.20	56.70	0.17	3.34	0.90	1.94	0.13	5.20
KDK_S.5	D	4.11	0.35	18.70	55.80	0.20	3.51	0.86	1.95	0.13	5.27
NBC_S.16	D	2.86	0.43	17.40	52.60	1.34	4.79	1.10	3.09	0.17	6.17
NBC_S.17	D	2.37	0.66	16.70	54.20	1.60	2.35	2.51	1.15	0.10	6.82
NBC_S.18	D	2.96	0.59	18.10	56.50	0.53	4.17	1.40	2.86	0.18	6.22
NBC_S.20	D	4.72	0.45	18.30	51.90	1.10	2.33	1.60	2.00	0.96	6.81
NBC_S.22	D	2.42	1.35	16.90	52.80	1.36	2.40	2.28	0.86	0.08	7.43
NBC_S.23	D	1.00	3.57	17.80	44.10	1.70	0.28	4.77	1.49	0.26	12.90
NBC_S.24	D	3.93	1.16	17.30	46.40	1.85	2.19	1.73	1.52	0.06	9.99

*n.d.* not detected.

The samples of the Kindoki Group recovered from the Kindoki site show a remarkable enrichment in magnesium oxides (8–10%), attributed to the presence of talc. This typological group presents a potassium oxide content in a 1.5 to 2.5% range and low sodium (< 0.2%) and calcium oxide (< 0.4%) concentrations.

The high concentration of iron oxides (7.5–9%) is a common attribute of Kongo Type A pots. The Kongo Type A samples from Mbanza Kongo and Kindoki exhibit higher concentrations of potassium (3.5–4.5%). The high magnesium oxide content (3–5%) differentiates the Ngongo Mbata samples from the rest of the same typological group. The Kongo Type A sample NBC_S.4 presents a remarkably high concentration of iron oxides related to the presence of amphibole mineral phases. The Kongo Type A sample NBC_S.3 displays a high manganese concentration (1.25%).

Silicon oxide (60–70%) dominates the Kongo Type C sample composition, inherent to the quartz amount identified by XRD and petrography. Low sodium (< 0.5%) and calcium (0.2–0.6%) contents were observed. The higher concentration of magnesium oxide (13.9 and 20.7%, respectively) along with lower iron oxide in the MBK_S.14 and KDK_S.20 samples is consistent with the abundant talc mineral phase. Samples MBK_S.9 and KDK_S.19 of this typological group present a lower silica concentration and higher sodium, magnesium, calcium and iron oxide contents following the presence of micas, amphiboles and plagioclases identified by petrography and XRD. The higher titanium oxide (1.5%) concentration distinguishes the Kongo Type C sample MBK_S.9.

A differentiation in the elemental composition is indicative of Kongo Type D samples, pointing to lower silica content, in the range 44 to 63% and relatively high concentrations in sodium (1–5%), calcium (1–5%) and potassium oxides (1–5%) attributed to the presence of feldspars. Moreover, a higher titanium oxide (1–3.5%) content is observed in this typological group. The high iron oxide content of Kongo Type D samples MBK_S.15, MBK_S.19 and NBC_S.23 is correlated with higher magnesium oxide content, which is consistent with the dominance of amphiboles. High manganese oxide concentrations were detected in all Kongo Type D samples.

**Figure 7:**
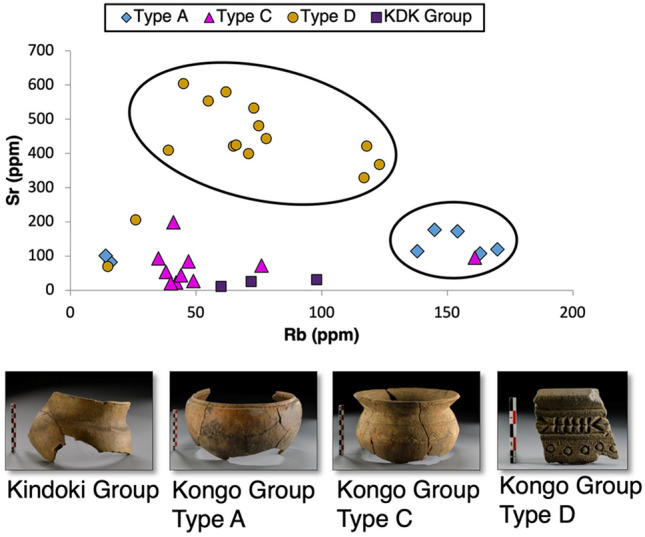
XRF data. Scatter plot Rb–Sr, selected samples from Kongo kingdom pots, colour-coded by typological group. Plot indicating the correlation between Kongo Type D pots and strontium and between Kongo Type A pots and rubidium.

The major element data suggest a correlation between calcium and iron oxides in the Kongo Type A and Type D pots, which is related to sodium oxide enrichment. Concerning minor elemental compositions (Supplement 6, Table S1), most of the Kongo Type D samples are enriched in zirconium, which shows a moderate correlation with strontium. The Rb–Sr diagram (Fig. 7) indicates an association between strontium and the Kongo Type D pots and another one between rubidium and the Kongo Type A pots. The Kindoki Group and the Kongo Type C ceramics are depleted in both elements. (See also Supplement 6, Figs. S16–S19).

**Figure 8:**
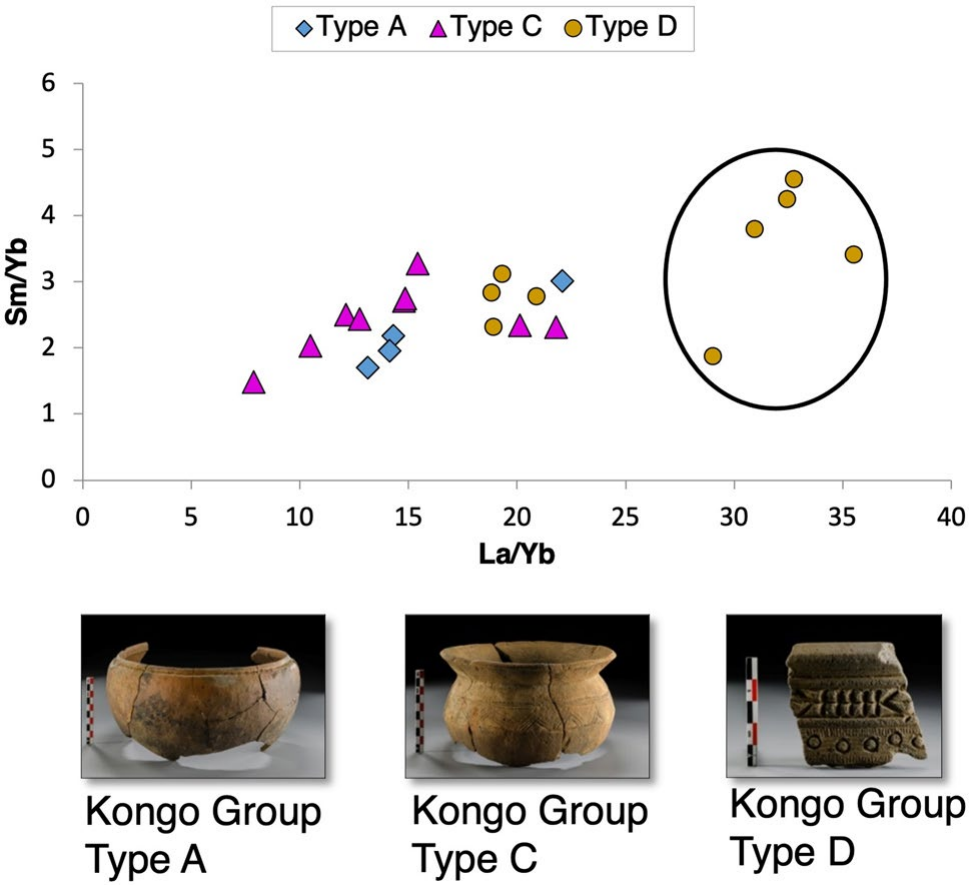
ICP-MS data. Scatter plot of La/Yb-Sm/Yb, selected samples from Kongo kingdom pots, color coded by typological group. The Kongo Type C sample MBK_S.14 is not depicted on the diagram.

Representative samples from Mbanza Kongo were analysed by ICP-MS to determine minor and trace elemental compositions and to investigate REE pattern distributions among the typological groups. The minor and trace elements are presented extensively in Supplement 7, Table S2. The Kongo Type A samples are enriched in thorium, as well as the Kongo Type D MBK_S.7, MBK_S.16 and MBK_S.25 samples. Kongo Type A pots present a relatively high concentration of zinc and are enriched in rubidium, while Kongo Type D pots present a high concentration of strontium, confirming the XRF results (Supplement 7, Figs. S21–S23). The La/Yb-Sm/Yb plot illustrates a correlation and depicts a high lanthanum content in the samples of Kongo Type D pots (Fig. 8).

**Figure 9:**
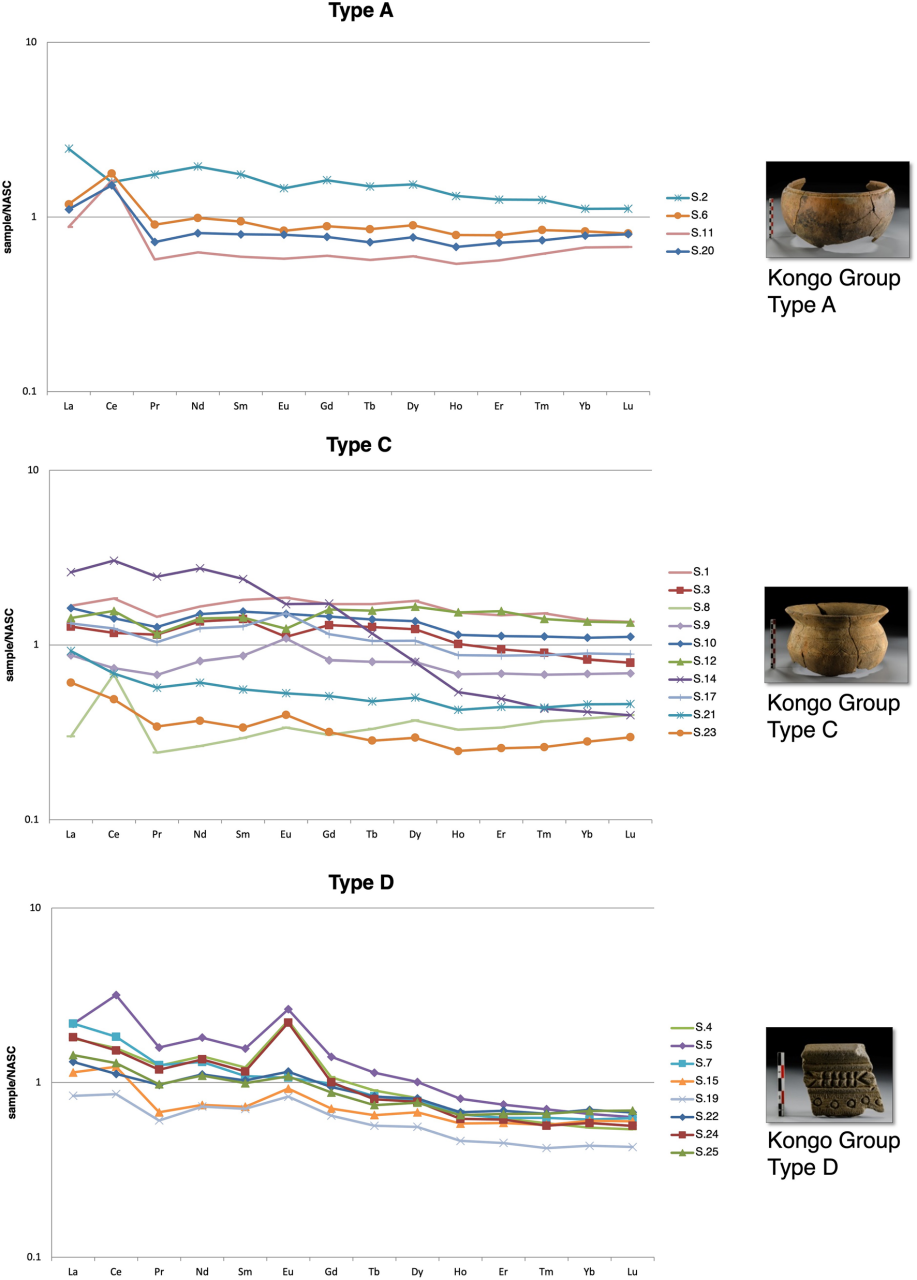
ICP-MS data. NASC-normalized REE diagram of the samples from Mbanza Kongo (MBK).

REE, normalized by NASC^47^, are presented in spider diagrams (Fig. 9). The results suggest an enrichment in light rare-earth elements (LREE), especially in the samples of Kongo Type A and Type D pots. Kongo Type C shows a higher variability. A positive europium anomaly characterizes Kongo Type D, and a high positive cerium anomaly characterizes Kongo Type A.

## Discussion

In this study, we examined a set of ceramics from three Central African archaeological sites linked with the Kongo kingdom and belonging to different typological groups, namely, the Kindoki and Kongo Groups^35^. The Kindoki Group represents an earlier period (early kingdom period) and is present only at the archaeological site of Kindoki. The Kongo Group—Types A, C and D—is concurrently present in the three archaeological sites. The Kongo Group is dated to the kingdom period. It represents an epoch during which contacts with Europe were established and goods were exchanged across and beyond the Kongo kingdom, as they had been for centuries. The compositional and petrofabric fingerprints were obtained using a multi-analytical approach. This is the first time such a protocol has been used in Central Africa.

The consistent compositional and petrofabric fingerprint of the Kindoki Group points to a distinct Kindoki production. The Kindoki Group is possibly linked to the period that Nsundi was an independent province of the Seven Kingdoms of Kongo dia Nlaza^28,29^. The presence of talc and vermiculite—a low-temperature product of talc weathering—in the Kindoki Group indicates the use of local raw materials, as talc is present in the geological substrate of the Kindoki site, in the *Schisto-Calcaire* Group formation^39,40^. The fabric features of this pot typology, observed through textural analysis, point to a non-advanced raw material processing.

The Kongo Type A pots show some intra- and inter-site compositional variations. The ones from Mbanza Kongo and Kindoki present high concentrations of potassium and calcium oxides, while those from Ngongo Mbata have a high magnesium content. However, some shared features differentiate them from the other typological groups. They are more consistent in the fabric, marked by a micaceous paste. They show a relatively high content of feldspars, amphiboles and iron oxides, unlike Kongo Type C. The high content of micas and the presence of tremolite amphiboles distinguish them from Kongo Type D pots, in which actinolite amphiboles were identified.

Kongo Type C also presents variations in the mineralogical and chemical compositions and in the fabric features in each of the three archaeological sites and across them. This variability is attributed to the exploitation of any available raw material source in the proximity of each production/consumption place. Nevertheless, stylistic similarity was achieved aside from local technological adaptations.

**Figure 10:**
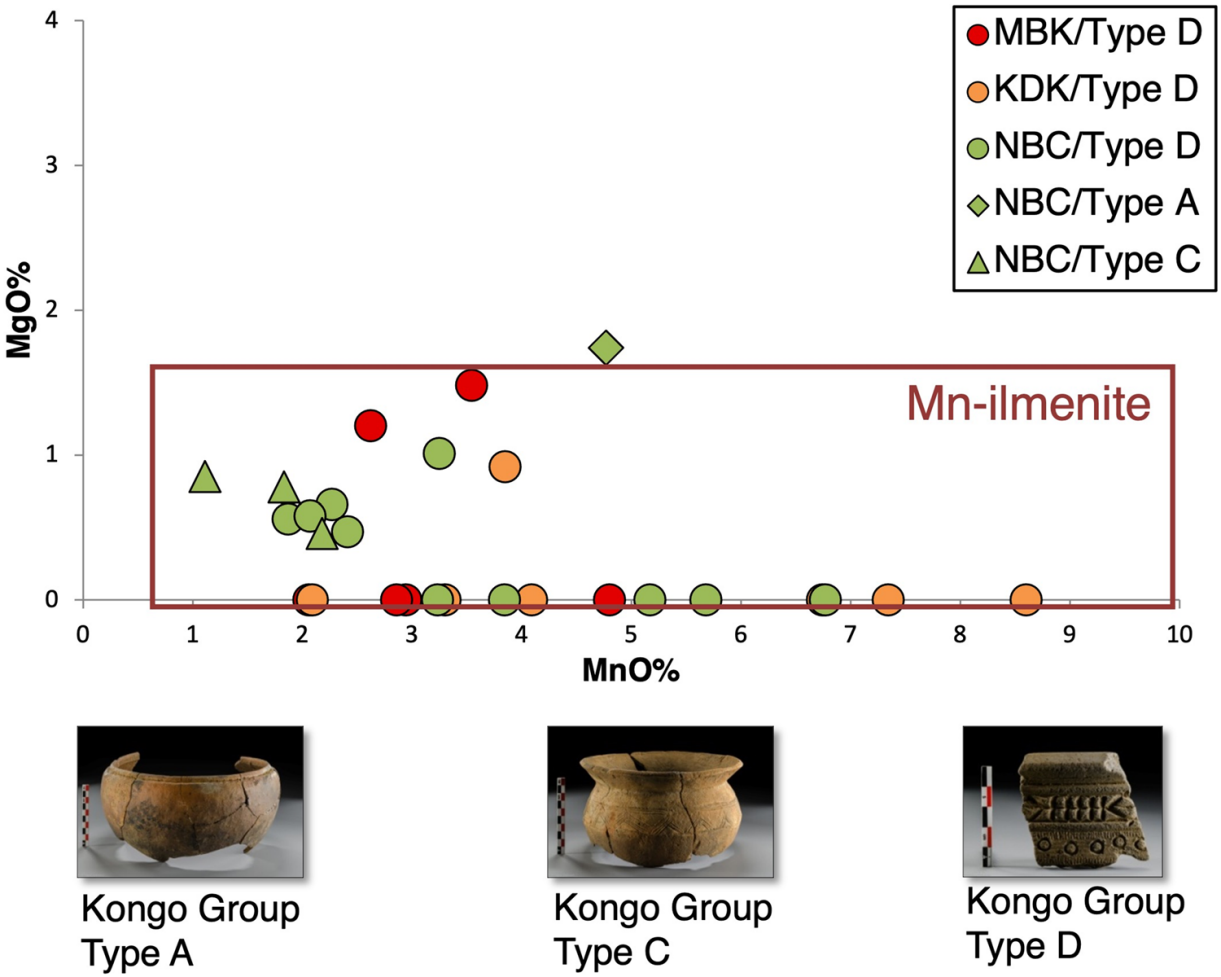
VP-SEM-EDS data. Scatter plot of MgO–MnO, selected samples with identified ilmenite grains from Mbanza Kongo (MBK), Kindoki (KDK) and Ngongo Mbata (NBC), indicating the manganoan-ilmenites (Mn-ilmenites) based on the study of Kaminsky and Belousova^49^.

Kongo Type D is strongly correlated with a high concentration in titanium oxides, attributed to the presence of ilmenite minerals (Supplement 6, Fig. S20). The high manganese content of the analysed ilmenite grains links them to manganoan-ilmenites (Fig. 10), a distinct composition compatible with kimberlite rock formations^48,49^. The presence of *Cretaceous* continental sedimentary rocks—a secondary sedimentary source of diamonds after the erosion of *pre-Cretaceous* kimberlite pipes^42^—and the reported Kimpangu kimberlite field^43^ in the Lower Congo suggest the broader region of Ngongo Mbata as a possible source of raw materials for Kongo Type D pottery production. This is further supported by the detection of manganoan-ilmenites in one Kongo Type A sample and one Kongo Type C sample from the Ngongo Mbata site.

The positive europium anomalies observed in the REE patterns (see Fig. 9) of Kongo Type D pots and especially in the samples with identified ilmenite grains (e.g., MBK_S.4, MBK_S.5 and MBK_S.24) could be correlated with ultrabasic igneous rocks enriched in Ca-feldspars that retain Eu^2+^. This REE distribution could also explain the high strontium concentration, identified in Kongo Type D samples (see Fig. 6), as strontium substitutes calcium in the Ca-mineral crystal lattice^50^. The high lanthanum content (see Fig. 8) and the general enrichment in LREE (see Fig. 9) could be attributed to ultrabasic igneous rocks as kimberlite-like geological formations^51^.

The particular compositional features of Kongo Type D pots that link them to a specific natural source of raw materials along with the inter-site compositional affinities of this typology suggest a singular production centre for Kongo Type D pots. In addition to the compositional peculiarities, the temper-grain size distribution of Kongo Type D results in a very hard ceramic artefact and indicates intentional raw material processing and advanced technological knowledge in pottery production^52^. This feature is unique and further supports the interpretation of this type as a product intended for a specific elite group of users^35^. Regarding this production, Clist et al.^29^ suggest that it was possibly the result of an interaction between Portuguese tile-makers and Kongo potters, as such a technological know-how was never encountered during and before the kingdom period.

The lack of newly formed mineral phases in the samples of all the typological groups indicates that low-temperature firing (< 950 °C) was applied, which is also in accordance with the ethnoarchaeological studies carried out in the region^53,54^. Furthermore, the absence of hematite and the dark color of some of the potshards are due to a reducing firing or due to post-firing^4,55^. Ethnographical research in the region has shown post-firing processing performance during pottery manufacture^55^. The dark colour, identified mostly in Kongo Type D pots, could be suggested as part of their rich decoration, linked to the targeted users. Ethnographical data in the broader African context support this statement, in that blackened pots are often attributed a specific symbolic significance^56^.

The low concentration of calcium across the samples, the absence of carbonates and/or their respective newly formed mineral phases are attributed to the non-calcareous nature of the ceramics^57^. This issue is particularly interesting for the samples enriched in talc (mainly the Kindoki Group and Kongo Type C pots) due to the concurrent presence of carbonates and talc phases in the local carbonate-pelite assemblage—the Neoproterozoic *Schisto-Calcaire* Group^42,43^. Intentional procurement of certain types of raw materials from the same geological formation indicates advanced technological knowledge related to the unsuitable behaviour of calcareous clays when they are fired at low temperatures.

In addition to the intra- and inter-site compositional and petrofabric variations of Kongo Type C pots, the high demands of cooking pot consumption allow us to place Kongo Type C pottery production at a community level. Nevertheless, the quartz content in the majority of the Kongo Type C samples points to some level of coherence in pottery production across the kingdom. It demonstrates a deliberate selection of raw materials and advanced technological knowledge related to the competent and suited function of quartz-tempered cooking pots^58^. Quartz-tempering and the absence of calcareous materials demonstrate that raw material selection and processing also relied on techno-functional demands.

The production of the same typology (Kongo Type C) at different archaeological sites provides evidence regarding the relative technical skills and knowledge of the different production centres^59^. In this respect, the more coherent ceramic culture of Ngongo Mbata in terms of composition and fabric implies a higher degree of specialisation.

The technological integrity within each typological group at Ngongo Mbata, along with a specialised pottery production (Kongo Type D), provides evidence of a competent production centre, with an activity that was uninfluenced by the internal socio-political fluctuations. Craft specialisation in Ngongo Mbata is further supported by the existence of stone tobacco pipe workshops, whose production first and foremost targeted Mbata’s elite^60,61^. Day^59^ highlights a correlation between craft specialisation and the creation and maintenance of ruling elites. Taking this into consideration, we can draw an intimate link between Ngongo Mbata’s specialised production and its elite status, which is in accordance with oral traditions and historical information, placing Mbata province in a more privileged position than the other provinces^17,28^.

## Conclusions

This study contributes to a further understanding of the cultural and trade networks in the Kongo kingdom. It enables us to investigate a complex society through the conceptual framework involved in natural resource exploitation and raw material procurement and processing. This approach is vital, especially with regard to the historical archaeology of Central Africa, where most information arises from oral traditions and few written sources, which are often shaped by a Eurocentric perspective.

The Kindoki Group is an earlier ceramic production (early-kingdom) than the Kongo Group. The various petrofabric features of these two groups show technological development in pottery production during the kingdom’s formation. Nevertheless, some degree of technical competence in the locally produced pots of the Kindoki Group is proven by the selection of specific technologically suitable raw materials from a complex geological formation. As this is also observed in the Kongo Type C pottery, we could assume some level of continuity of the pottery tradition throughout the kingdom period. The existence of Kongo Type C pottery production at a community level across the kingdom is proven by the intra- and inter-site compositional and petrofabric variations identified. Nevertheless, the fact that Kongo Type C pottery shares techno-functionally significant features (quartz-tempering and absence of calcareous materials) on an inter-site scale implies the transmission of knowledge from one production centre to the other and points to some level of cohesion of pottery-making traditions across the kingdom. Additional evidence for this interpretation is that the inconsistency in raw material sourcing does not result in any stylistic differentiation.

Our results suggest that Mbata Province was the unique production centre for Kongo Type D pots, which underlines its importance in the wider socio-political framework of the Kongo kingdom. From there, these pots circulated across the kingdom. The deliberate procurement of raw materials and their cautious processing for the manufacturing of these prestige objects, in addition to the evidence for product specialisation, reveal possible social preferences. The compositional affinities between Kongo Type A and Kongo Type D could be seen as an attempt of Kongo Type A potters to imitate Kongo Type D production. However, fabric and compositional consistency within Kongo Type A pots supports the existence of production templates throughout the kingdom.

The relatively diverse mineralogical and elemental signature of the Mbanza Kongo pots can be attributed to the different provenances of ceramic artefacts, underlining Mbanza Kongo’s key position at a major crossroad of the trade routes as the capital of a centralised polity.

Pottery production in the Kongo kingdom reflects a model of political economy, where material culture subjected to specific patterns of consumption is circulated, distributed and redistributed under the control of a central power. Pottery traditions and pottery circulation in the kingdom reflect well-established interaction and exchange networks. Long-distance distribution of high-quality objects (Kongo Type D), competent pottery production centres associated with, if not controlled by the elite, and community production centres that follow stylistic patterns and techno-functional demands (Kongo Type A and Kongo Type C) constitute inherent elements of centralisation and social complexity. These conclusions tie in with the qualification of the Kongo Kingdom as a centralized polity ruled by an elite.

## Methods

Shards of 67 ceramic pots, dated to the period between the fourteenth and eighteenth centuries AD, from the excavations in Mbanza Kongo (Angola) and in Kindoki and Ngongo Mbata (Democratic Republic of Congo), were selected for the analysis. The ceramic fragments were initially sub-sampled and prepared for the analysis following the standard procedure (for the whole sample preparation procedure, see Supplement 8).

### Material characterization

The samples were analyzed by X-ray diffraction (XRD), thermogravimetric analysis (TGA), petrographic analysis, variable pressure scanning electron microscopy coupled to energy dispersive X-ray spectroscopy (VP-SEM-EDS), X-ray fluorescence spectroscopy (XRF) and inductively coupled plasma mass spectrometry (ICP-MS). The multi-analytical approach allows us to deal with these complex sample-sets.

The bulk mineralogical composition was obtained using a Bruker D8 Discover X-ray Diffractometer with a Cu Kα source operating at 40 kV and 40 mA and a LYNXEYE linear detector. The diffractograms were collected at a 2θ angular range of 3°–75°, with a 0.05° step size and 1 s measuring time by point. The reference intensity ratio (RIR) method^62^ was used for the semi-quantitative determination of the mineral phases, providing the mineral abundance in the bulk samples as a percentage relative to the presumed 100% matrix of crystalline minerals. Oriented aggregate mounts for clay-mineral identification were analysed at a 2θ angular range of 3°–75°, with a 0.05° step size and 1 s measuring time by point. The clay-minerals were identified according to the U.S. Geological Survey (USGS) clay mineral identification flow diagram^63^.

Specific mineral phases were identified by thermogravimetric analysis using a Netzsch STA 449F3 Jupiter analyzer. The selected samples were heated in Pt-Ir crucibles from 40 up to 1000 °C with a heating rate of 10 °C/min under a nitrogen atmosphere.

The ceramic petrography was performed using a Leica DM2500P transmitted light polarising microscope in both plane-polarised light (PPL) and cross-polarised (XP) modes for the mineralogical and textural characterisation of the samples. The microscope is coupled with a Leica MC 170HD digital camera for image acquisition.

The major elemental compositions of SiO_2_, TiO_2_, Al_2_O_3_, Na_2_O, K_2_O, CaO, MgO, MnO, FeO, and P_2_O_5_ were obtained by a Bruker S2 Puma Energy Dispersive X-ray Spectrometer (EDS-XRF) spectrometer equipped with a silver anode X-ray tube (software: Spectra Elements 2.0), using a methodology described elsewhere^64^. The quantitative data were obtained using a regression method with 19 siliceous standard reference materials (SRMs) from USGS SRM: GSP-2, SBC-1, BCR-2, BHVO-2, BIR-1A, DTS-2B, SGR-1B, SDC-1, QLO-1, AVG-2, COQ-1, MINTEK SRM: SARM-52, STSD-3 Natural Resource Canada SRM: STSD-3, LGC SRM: SXO7-10, and NCS SRM: DC 60105, DC 73028, DC 61101, DC 62108c, DC 73309.

Microanalysis of the samples was performed by a HITACHI S-3700N variable pressure scanning electron microscope operated with an accelerating voltage of 20 kV and a chamber pressure of 40 Pa and a Bruker XFlash 5010 silicon drift detector (SDD) with a resolution of 129 eV at Mn Ka. The EDS elemental data were acquired by point microanalysis and in the form of elemental distribution maps. The SEM images were captured in backscattering (BSE) mode.

Minor and trace element compositions were obtained using an Agilent 8800 ICP-MS Trip Quad system. All reagents used were of suprapur or OPTIMA grade. For the preparation of standard solutions, ultrapure water (18.2 MΩcm, Milli-Q, Millipore Integral 3, Darmstadt, Germany) and nitric acid Suprapur grade (65.0%, Merck) were used. The equipment was calibrated according to the standard calibration procedure with an Agilent Technologies tuning solution. The ICP-MS tuning solution used contains 10 μg/L each of Ce, Co, Li, Tl, and Y in a 2% HNO_3_ matrix (Agilent Technologies, Palo Alto, CA, USA). Prior to the analysis, equipment sensitivity was optimised, and oxide formation (< 1.2%) and double charged ions (< 2%) were minimised. The analysis was performed in spectrum mode, and the collision/reaction cell was in no-gas, He, O_2_, and NH_3_ modes according to element features. All the operation modes were with the MS/MS mode scan type. The analysis was optimised at 1550 W radio-frequency power and 1.01 L/min carrier gas flow (Ar). The plasma gas flow (Ar) rate was 15 L/min, and the reaction gas flow (He, O_2_, NH_3_) rates were 4 mL/min, 0.5 mL/min and 1.5 mL/min, respectively. Each sample was measured in triplicate with 10 sweeps per replicate, and the relative standard deviation (RSD) was reported.

The following analytes of interest were, for minor elements with selected masses at Q1/Q2: 45/45 (Sc), 51/51 (V), 59/59 (Co), 60/60 (Ni), 63/63 (Cu), 66/66 (Zn), 71/71 (Ga), 72/72 (Ge), 85/85 (Rb), 88/88 (Sr), 89/89 (Y), 90/90 (Zr) and 93/93 (Nb) and, for trace elements with selected masses at Q1/Q2: 133/133 (Cs), 137/137 (Ba), 139/139 (La), 140/140 (Ce), 141/141 (Pr), 146/146 (Nd), 147/147 (Sm), 153 (Eu), 157/157 (Gd), 159/159 (Tb), 163/163 (Dy), 165/165 (Ho), 166/166 (Er), 169/169 (Tm), 172/172 (Yb), 175/175 (Lu), 178/178 (Hf), 181/181 (Ta), 182/182 (W), 208/208 (Pb), 209/209 (Bi), 232/232 (Th) and 238/238 (U). The general instrumental conditions, the analytes of interest and their integration times are illustrated in Supplement 9, Table S3. For the quantification of the analytes of interest, the external calibration method was applied, and a calibration curve was built using multi-elemental standards (ICP-MS-68-A and ICP-MS-68-B; High Purity Standards, Charleston, SC, USA) in a matrix of 2% HNO_3_. The calibration curve was made of 8 different levels with concentrations: 0, 50, 100, 200, 400, 800, 1600 and 3000 ppb. Ru, Rh, and Ir were added online along the measurements and used as internal standards to correct possible instrumental drifts and matrix effects. Two certified reference materials (CRMs) from the United States Geological Survey (USGS) (CRMs: AGV-2, Guano Valley Andesite and W2-a, Centerville Survey) were measured after each set of 10 samples to evaluate the quality of the data and to validate the analytical method. CRMs and sample digestion blanks were included in all analytical runs.

The limit of detection (LoD) was experimentally performed by measuring 11 replicates of a blank solution and of a 200 ppb standard solution. The LoD was calculated using the standard deviation of the 11 blanks (σ^*blank*^) applied in the following equation: LoD = 3σ^*blank*^200/(CPS^*200ppb*^-CPS^*blank*^). The limit of quantification (LoQ) was calculated as LoQ = 10LoD. The selected elements, their respective collision/reaction gas mode along with the detection and quantification limits (LoD and LoQ), presented in ppb (parts per billion), are given in Supplement 9, Table S4. The detected levels for tantalum are below the LoDs and thus are not included in the results. Germanium, tungsten and bismuth are not certified.

The research methodology was designed, applying a multi-analytical approach, to collect complementary data relevant to the research questions listed above. XRD, applied to all samples to identify and semi-quantify their bulk mineralogical compositions, was used as a primary technique in this study to cluster the studied samples. Three representative samples were analyzed by TGA to distinguish between pyrophyllite and talc, as their XRD profiles are similar. Petrography was applied to all samples to provide additional information regarding the mineralogy of the temper and the petrofabric of the ceramic material. Six of the samples (4 Kongo Type C and 1 Kindoki Group) were not analysed by petrography either due to the limited amount of sample or due to their high quartz content. Representative samples were analyzed by VP-SEM-EDS, focusing on the composition of the ilmenite grains and of the feldspars and amphiboles. XRF was applied to representative samples to determine their major element compositions. ICP-MS was implemented on the whole sample-set from Mbanza Kongo to provide minor and trace elemental compositions and to investigate REE patterns (Supplement 10, Table S5).

## Supplementary Information

Supplementary Information 1.

Supplementary Information 2.

## Data Availability

The materials used and the details on the methodology followed during the research as well the datasets produced during the analysis are available from the corresponding author on reasonable request.
