# Supplementary material for: A multi-analytical characterization of fourteenth to eighteenth century pottery from the Kongo kingdom, Central Africa
Source: Sci Rep. 2022 Jun 15;12:9943. doi: 10.1038/s41598-022-14089-x (PMC9200768; doi:10.1038/s41598-022-14089-x)
Supplement: Supplementary file 1 — Supplementary Information 1. [file 41598_2022_14089_MOESM1_ESM.pdf]

SUPPLEMENTARY INFORMATION

A multi-analytical characterization of fourteenth to eighteenth century pottery from the Kongo kingdom, Central Africa

Anna TSOUPRA[1], Bernard CLIST[2], Maria da Conceição LOPES[3], Patricia MOITA[1], Pedro BARRULAS[1], Maria da Piedade de JESUS[4], Sónia da Silva DOMINGOS[5], Koen BOSTOEN[6], José MIRAO[1,7*]

[1.]HERCULES Laboratory, University of Évora, Palácio do Vimioso, Évora, Portugal. atsoupra@gmail.com, pmoita@uevora.pt, pbarrulas@uevora.pt, jmirao@uevora.pt

[2] Institut des Mondes Africains (IMAF), Paris, France. bernard.clist@gmail.com

[3.]Research Center in Archaeology, Arts and Heritage Sciences, University of Coimbra, Coimbra, Portugal. conlopes@ci.uc.pt

[4.]Ministério da Cultura, Rua do MAT Complexo Administrativo, "Clássico do Talatona", Luanda, Angola. piedadejesus65@gmail.com

[5.]Centro Nacional de Investigação Científica (CNIC), , Luanda, Angola. tasonia2000@yahoo.fr

[6] Department of Languages and Cultures, BantUGent – UGent Centre for Bantu Studies, Ghent University, Ghent, Belgium, Koen.Bostoen@UGent.be

[7.]Department of Geosciences, School of Science and Technology, University of Évora, Colégio Luís António Verney, Évora, Portugal.

* Correspondence to:

José Mirão: jmirao@uevora.pt. HERCULES Laboratory, University of Évora, Largo Marquês de Marialva 8, 7000-809 Évora, Portugal. Phone number: +351 266 740 800

Supplement 1. The Kongo kingdom, Historical information

Based on the reconciliation of oral traditions, Thornton [17,18] proposes the starting point of the Kongo kingdom to be 1350-1375 AD, and suggests that Mbanza Kongo as was conquested by the kingdom's founders. Since its foundation until the late 17th century, the Kongo kingdom expanded significantly by the integration of new provinces, which were ruled by royal members under the direct administration of the king, except for the Mbata province and in later times the Soyo province [15,17,18,31]. The Kongo kingdom's social structure is characterised by a distinction between towns (*mbanza*) and villages (*mavata*) [15,17-20,30,31]. Towns were populated by the rulers, the nobles, and the slaves; the slaves contributed to the increase of population in the towns, a crucial feature of the kingdom's economy [19,27,30]. The villages, most abundant in the kingdom, were populated by commoners, most often of local descent [19]. This social division was further extended to the economic processes within the kingdom which had a centralised agricultural system. The management of production (land use, labor, and distribution) and taxation (either in kind, production surplus, specialised products of the rural regions or in money, such as nzimbu shells, palm cloth) also point towards a high degree centralisation within the kingdom [19,20,30].

Exchange of indigenous products stimulated the exploitation, production and manufacture of resources. Thus, the trade network was established on the basis of the local availability and Mbanza Kongo was the hub of this royal monopoly [20]. The expansion of the trade was an outcome of the contact with Portuguese in the late 15th century and was furthered by the existence of a common currency. Based upon the pre-contact networks, a long-distance trade was established, and transatlantic contacts were developed [19-22,25]. Subsequently, European material culture entered into the exchange system [19,20,24]. Another outcome of the Portuguese contact was the introduction and spread of Christianity [20,25]. The Christian religion was adapted to the existent religion and was established as a royal cult [20,25]. The conversion of the kingdom's elite to Christianity reinforced its relationships with Portugal [25].

It was only in the first half of the 17th century that the trade patterns were changed and the use of the trade routes that pass through Mbanza Kongo was reduced due to the outbreak of civil wars in 1665 [19,20]. These changes and their further effects, such as the evolution of Soyo into an independent province in 1636, the development of alliances between clans, and between provinces from inside and outside of the kingdom (Kimbazu and Kinlaza), as well as the increase of their control over regions, had an adverse impact on the political stability of the kingdom. The reduction of the central power resulted in a decrease of the population's mobility from the towns to the rural regions and the end of the central administration [19,20,22,27,30]. The gradual decline of Mbanza Kongo and of the kingdom led to its gradual incorporation into the Portuguese colony of Angola during the 19th century, marked by the signature of its vassalage treaty to the king of Portugal in 1861 [15,19].

Supplement 2. Archaeological information

Mbanza Kongo [66,67] The archaeological site of Mbanza Kongo (06° 16’ 080 S; 14° 14’ 590 E) is on a hilltop of 570m height, in the province of Zaire, in north-western Angola. The site was the capital of the Kongo kingdom. It was excavated in three campaigns in 2011, 2013 and 2014 by a collaboration of Angolan, Portuguese and Cameroonian archaeologists, within the framework of the project of the Angolan Ministry of Culture for the inscription of the site on the World Heritage list. The excavations were carried out in seven different archaeological stations Bairro Madungu, Tadi dia Bukikwa, Mpindi a Ntadi, Lumbu, Bairro Alvaro Buta, Kulumbimbi, and the Catholic Mission. A survey permitted on an eighth station to document a thick wall of 42,40m length, constructed of laterite blocks, which could be interpreted as the protection wall of the royal palace compound or of a church. The human occupation of the area, as evidenced by archaeology, ranges from the end of the 15th to the 19th century. Excavations identified part of a settlement dated to the 16th century (Bairro Madungu), an architectural structure that could be interpreted as the Jesuit convent dated to the first half of the 17th century (Tadi dia Bukikwa), an area, dated to the 17th century, interpreted as the fumigation place of the kings (Mpindi a Ntadi), a stone building that according to oral traditions, is associated with the royal gathering place and is dated to the second half of the 17th century and the 19th century) (Lumbu), part of a cemetery with several graves studied, probably associated with a church, in use in the 19th century (Bairro Alvaro Buta), the Mbanza Kongo cathedral and several burials dated to the 19th century with children graves outside of the church and a tomb of a young woman inside of it (Kulumbimbi) and at last a living area within the Catholic Mission dated to the 17th-19th century.

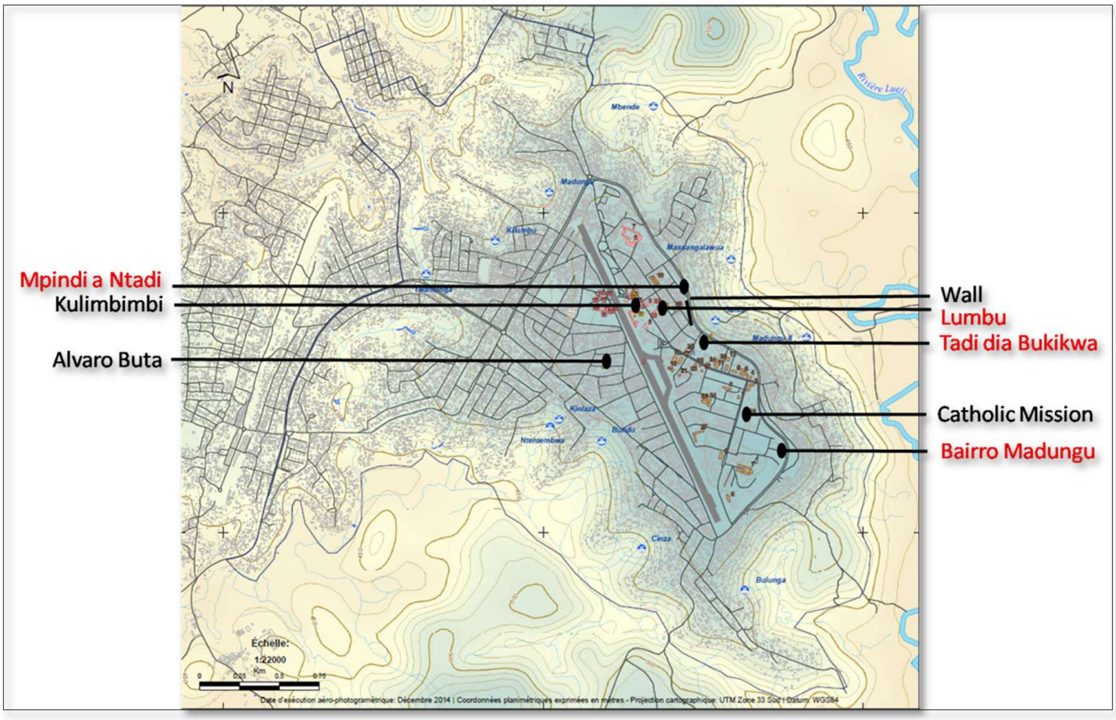

Figure S1. The eight archaeological stations in Mbanza Kongo, the locations of the examined potshards are in red (© B. Clist).

Kindoki [30,35,36] Kindoki archaeological site (05° 04’ 069 S; 15° 01’ 403 E) is located on a hilltop of 580 m height, in the eastern part of the Kongo Central Province of the DRC. The site is associated with Mbanza Nsundi, the capital of Nsundi province. The archaeological excavations, carried out on the Kindoki hill in three campaigns between 2012 and 2015 by the Kongoking project, uncovered an area of 537 m^2, following mainly a 50-m grid system of 1m^2 squares. The human occupation of the area ranges from the 14th to the early 20th century. Excavations in Kindoki revealed three settlements (dated to the 14th century, 16th-17th centuries and late 17th to the 20th century) and a cemetery, dated from the late 17th to the early 19th century, with eleven graves constructed and oriented the same way, and very closely set up one to the other. Their position together with the funerary goods is interpreted as being burials of the nobles and their associated kinships. Kindoki, as it is confirmed by radiocarbon dating, displays an evidence of continuous occupation from the 14th to the 20th century.

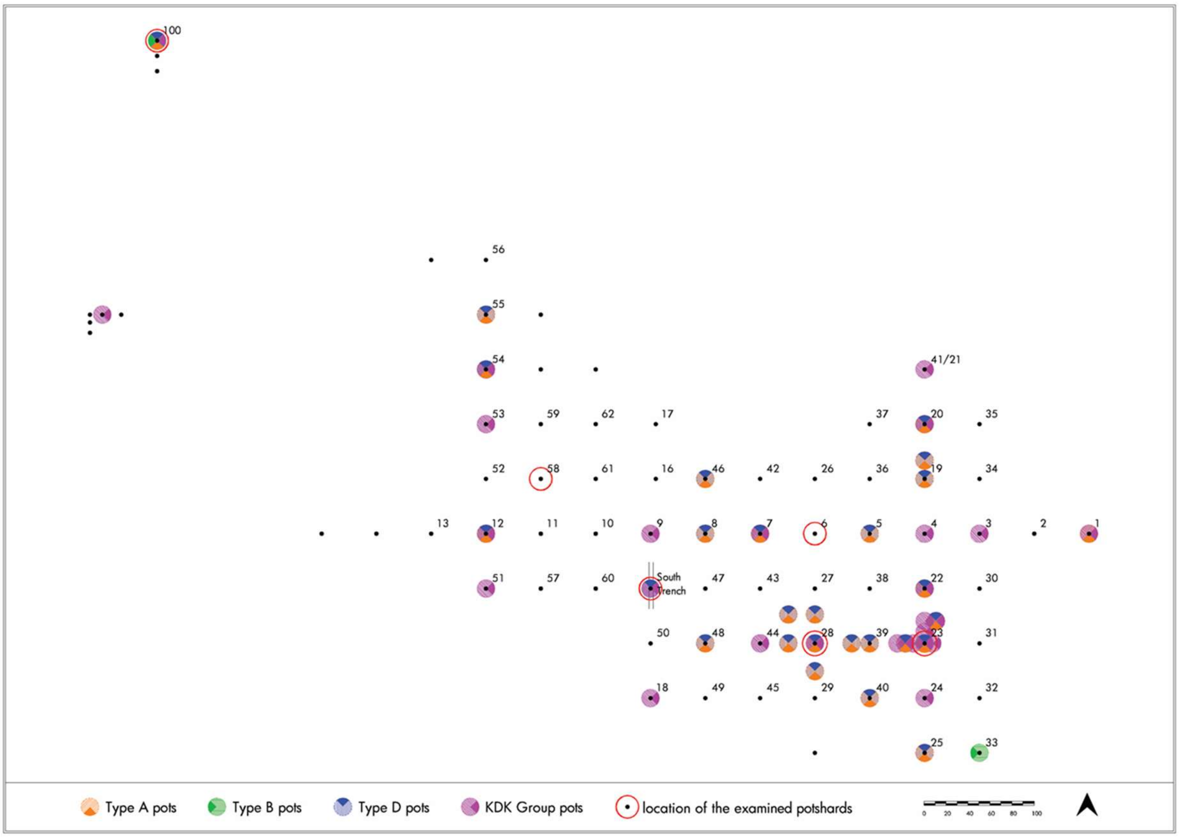

Figure S.2. Outline of the archaeological site of Kindoki (KDK), distribution of pots of the Kindoki Group and the Kongo Group, Type A, Type B and Type D, indicating the trenches of the examined shards. Kongo Type C potshards were found in high numbers in all trenches and units. Data from Kongo Type B pots are not presented here, as their sample size is not representative. (© B. Clist, modified by M. Triantafyllidou).

Ngongo Mbata [31,32] The archaeological site of Ngongo Mbata (05° 47′ 081 S; 15° 07′ 026 E) is located on a hilltop of 823 m height, north of the DRC-Angolan border. The site of Ngongo Mbata is linked historically with the city of Ngongo Mbata or Gongo de Batta, a distinct town of Mbanza Mbata. The first archaeological research on Ngongo Mbata hilltop was carried out between 1938-1942, undertaken by a Belgian archaeologist and priests. Recently, four archaeological campaigns were carried out between 2012 and 2015 by the Kongoking project. During the excavations an area of 847.5 m^2 was uncovered, following mainly a 50-m grid system of 1m^2 squares. The human occupation of the area ranges from the 16th to the late 18th century, with a high peak in the 17th century. Archaeological research in Ngongo Mbata (1938-1942) revealed a stone building, interpreted as the church of Ngongo Mbata (built between 1633 and 1667, recent radiocarbon dating), and its associated cemetery set up inside. Further excavations (2012-2015) in the area revealed several structures, including the foundations of a small house, possibly attributed to the priest's residence, an iron production area dated to the late 16th century, a stone smoking pipe workshop area, a stone structure, probably supporting a large wooden cross and the remains of three open-air cemeteries, in use during the 17th and 18th centuries. The church cemetery contained high-ranking burials as indicated by the funerary goods. Ngongo Mbata, as it is confirmed by radiocarbon dating, demonstrates that human occupation in the area was dominant from the 16th to the late 18th century, with a high peak in the 17th century.

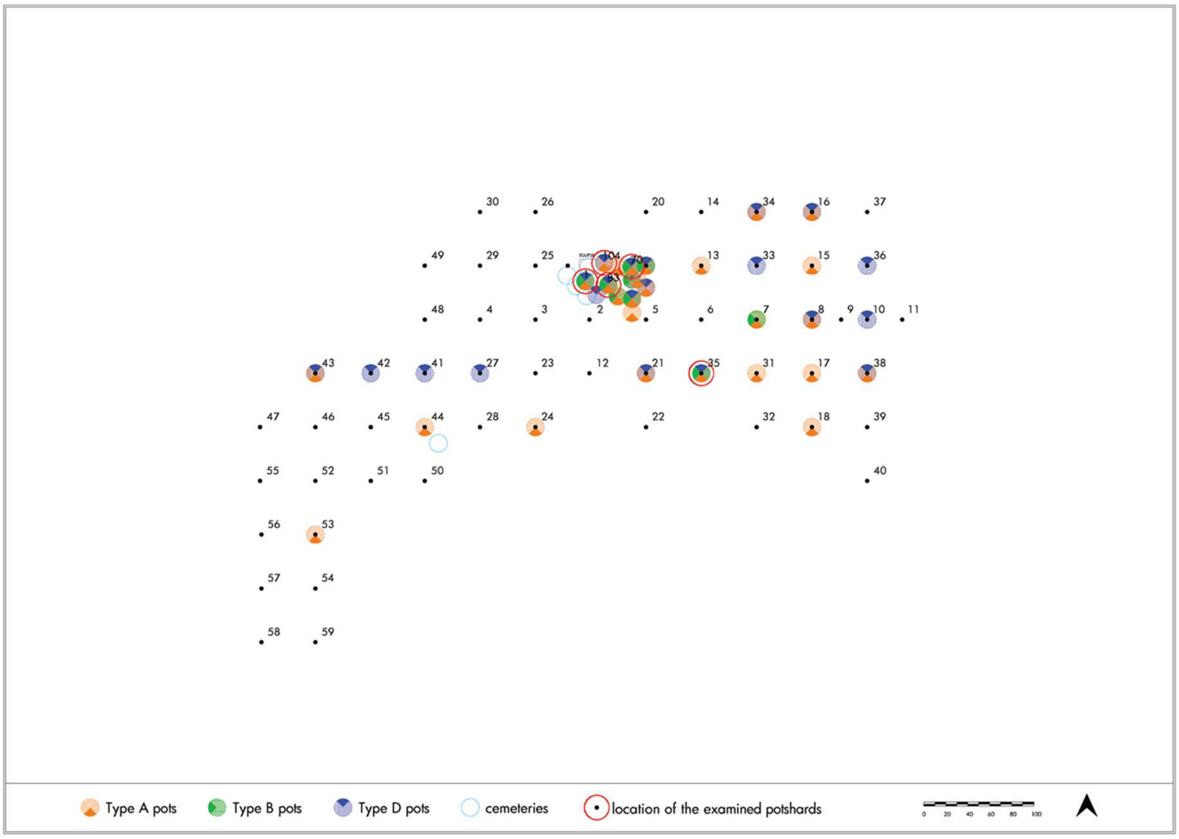

Figure S3. Outline of the archaeological site of Ngongo Mbata (NBC), distribution of pots of the Kongo Group, Type A, Type B and Type D, indicating the trenches of the examined shards. Kongo Type C potshards were found in high numbers in all trenches and units. Data from Kongo Type B pots are not presented here, as their sample size is not representative. (© B. Clist, modified by M. Triantafyllidou).

Supplement 3. ThermoGravimetric Analysis (TGA)

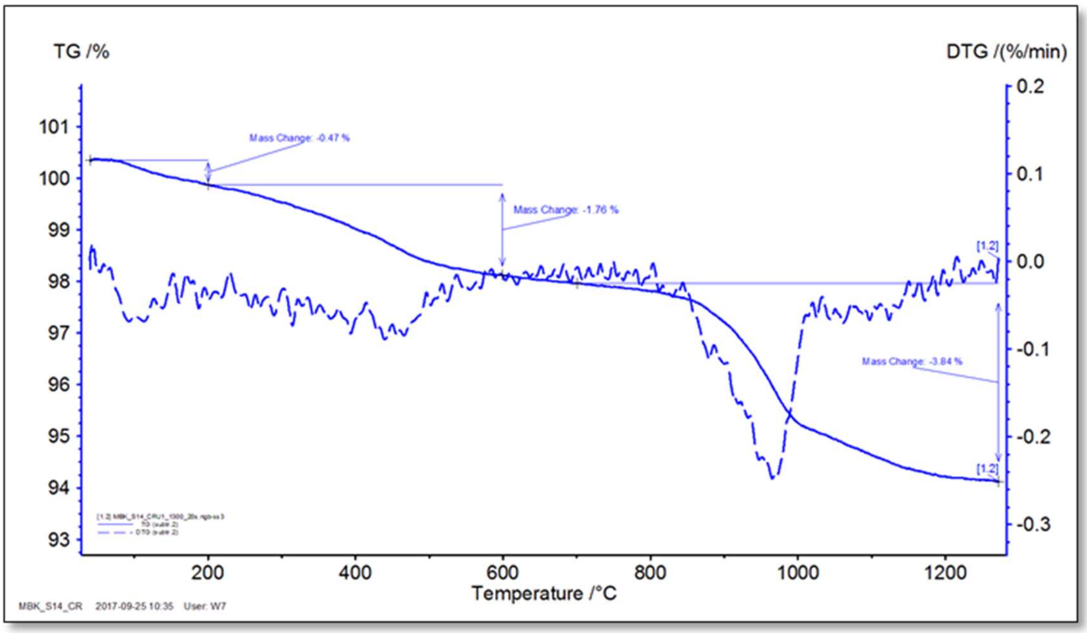

Figure S6. TGA results. TG curve of the sample MBK_S.14 (Kongo Group, Type C) illustrating the dehydroxylation and structure decomposition of talc between 850°C and 1000°C.

Supplement 4. Petrographic analysis

Figure S7. Optical microphotographs (2.5x) of the examined thin-sections from Mbanza Kongo (MBK), Kindoki (KDK) and Ngongo Mbata (NBC); colour coded by petrographic group: purple Group PGa, orange Group PGb, red Group PGc, green Group PGd and blue Group PGe.

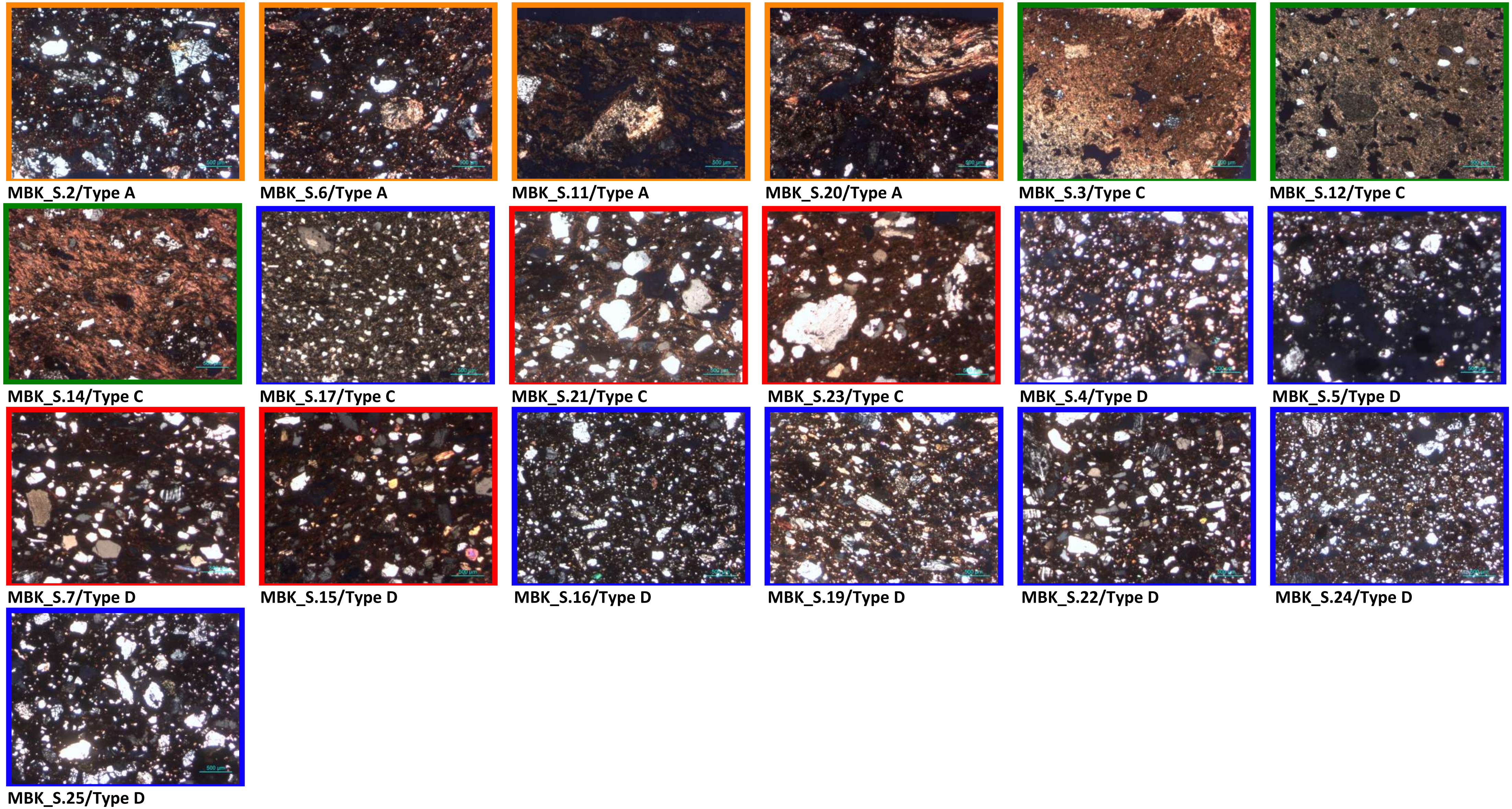

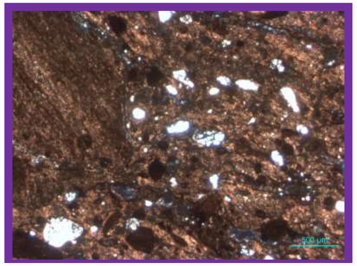
KDK_S.11/ KDK Group

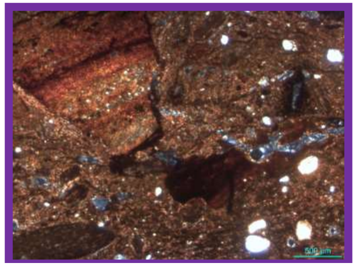
KDK_S.12/ KDK Group

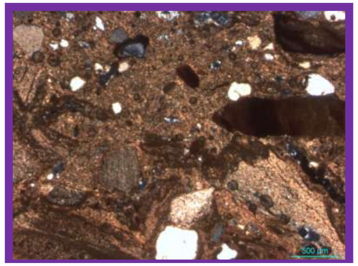
KDK_S.13/ KDK Group

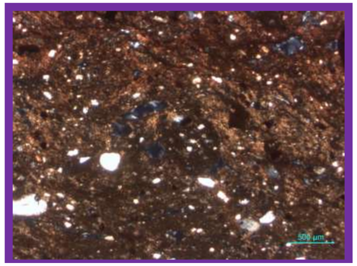
KDK_S.14/ KDK Group

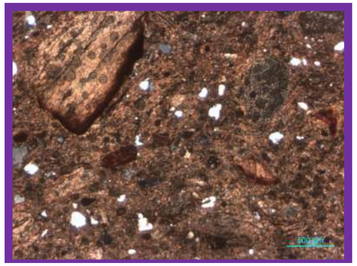
KDK_S.16/ KDK Group

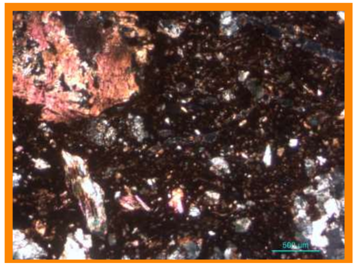
KDK_S.6/Type A

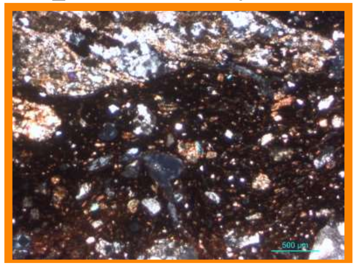
KDK_S.7/Type A

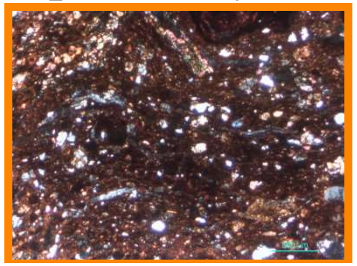
KDK_S.8/Type A

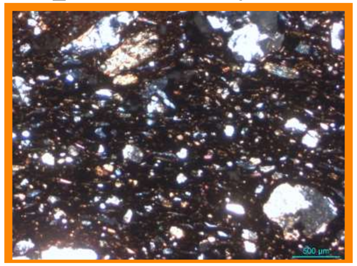
KDK_S.9/Type A

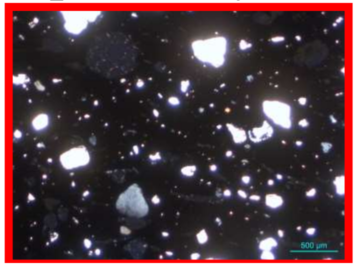
KDK_S.17/Type C

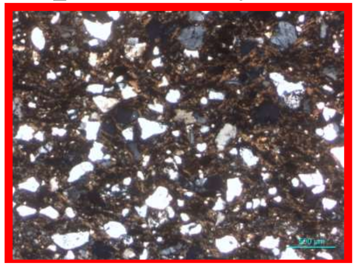
KDK_S.18/ Type C

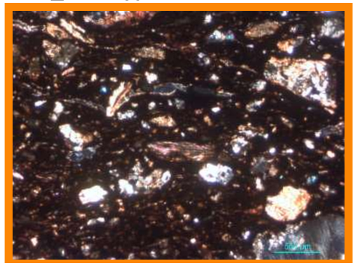
KDK_S.19/Type C

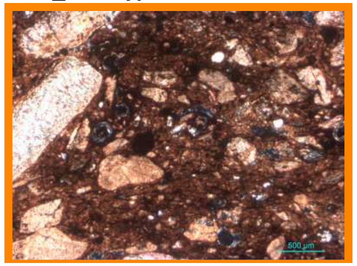
KDK_S.20/Type C

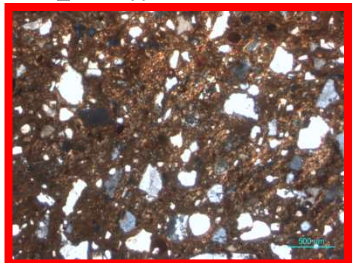
KDK_S.21/Type C

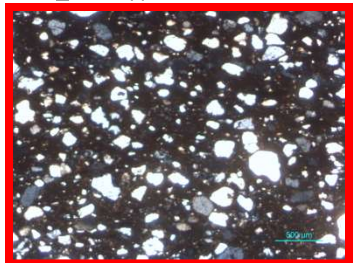
KDK_S.22/Type C

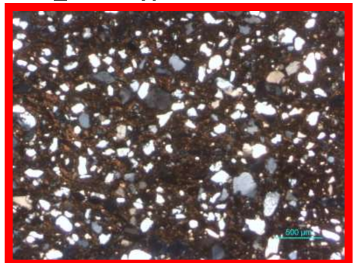
KDK_S.23/Type C

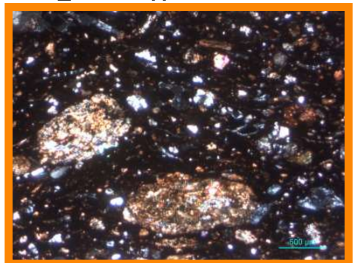
KDK_S.25/Type C

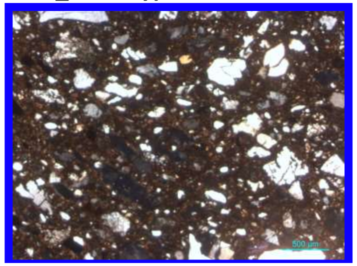
KDK_S.1/Type D

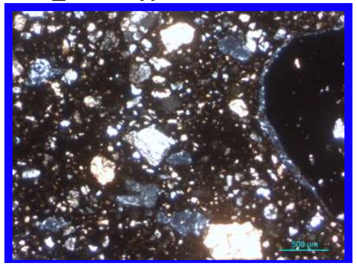
KDK_S.2/Type D

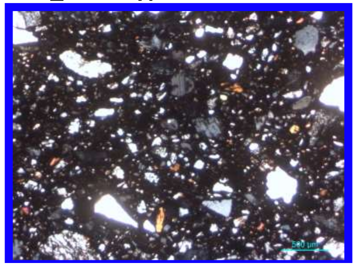
KDK_S.3/Type D

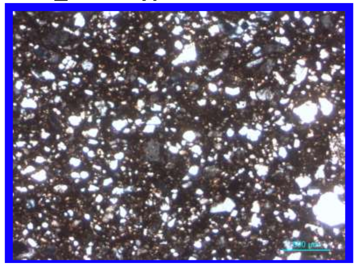
KDK_S.4/Type D

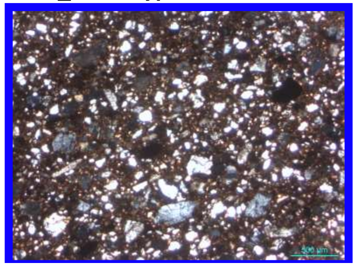
KDK_S.5/Type D

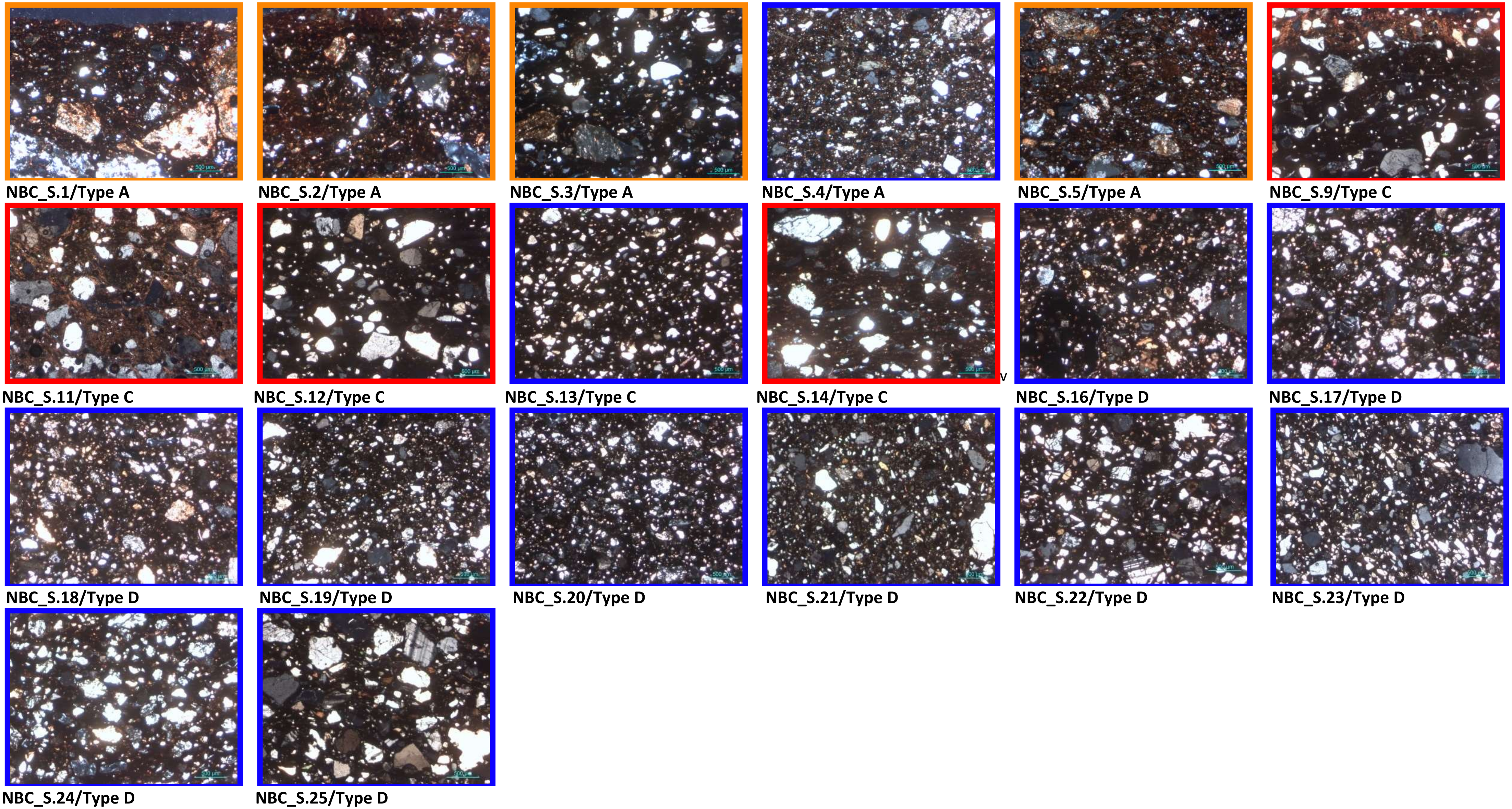
NBC_S.1/Type A
NBC_S.2/Type A
NBC_S.3/Type A
NBC_S.4/Type A
NBC_S.5/Type A
NBC_S.9/Type C
NBC_S.11/Type C
NBC_S.12/Type C
NBC_S.13/Type C
NBC_S.14/Type C
NBC_S.16/Type D
NBC_S.17/Type D
NBC_S.18/Type D
NBC_S.19/Type D
NBC_S.20/Type D
NBC_S.21/Type D
NBC_S.22/Type D
NBC_S.23/Type D
NBC_S.24/Type D
NBC_S.25/Type D

Supplement 5. Variable Pressure – Scanning Electron Microscope – Energy Dispersive X-ray Spectroscopy (VP-SEM-EDS)

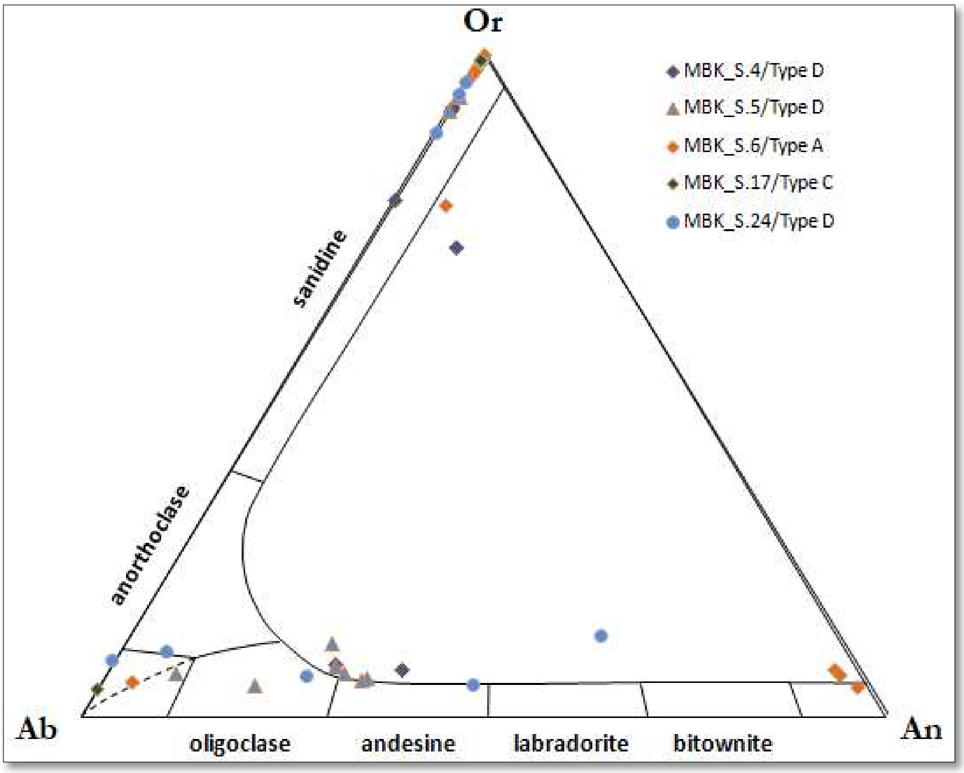

Figure S8. SEM-EDS data. Ternary diagram illustrating the composition of feldspars on selected samples from Mbanza Kongo (MBK), colour coded by sample.

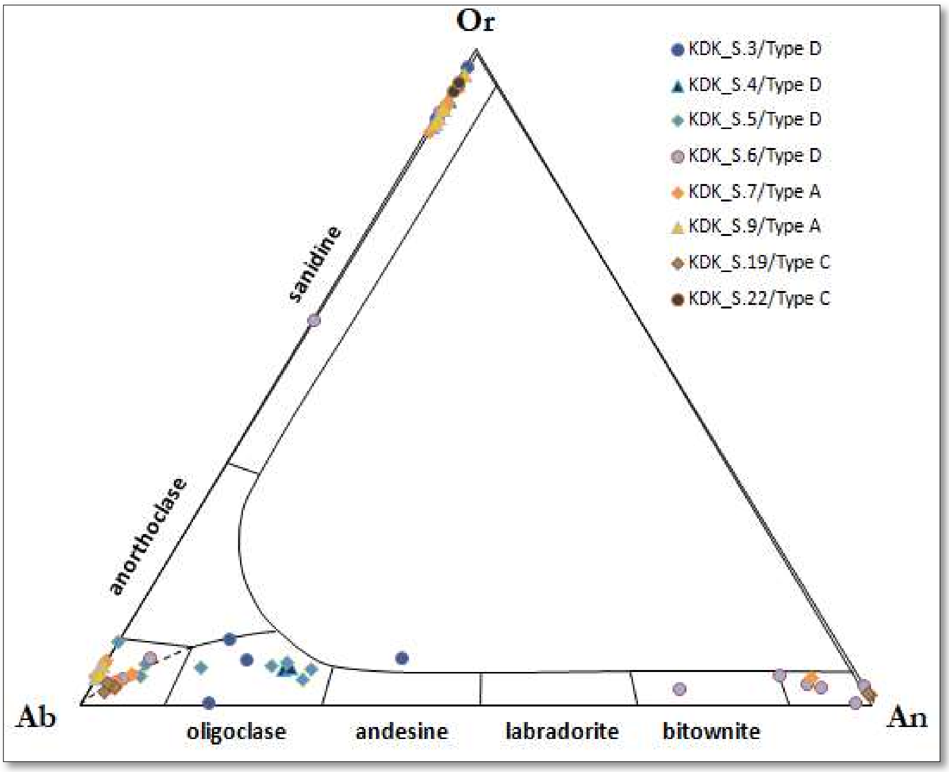

Figure S9. SEM-EDS data. Ternary diagram illustrating the composition of feldspars on selected samples from Kindoki (KDK), colour coded by sample.

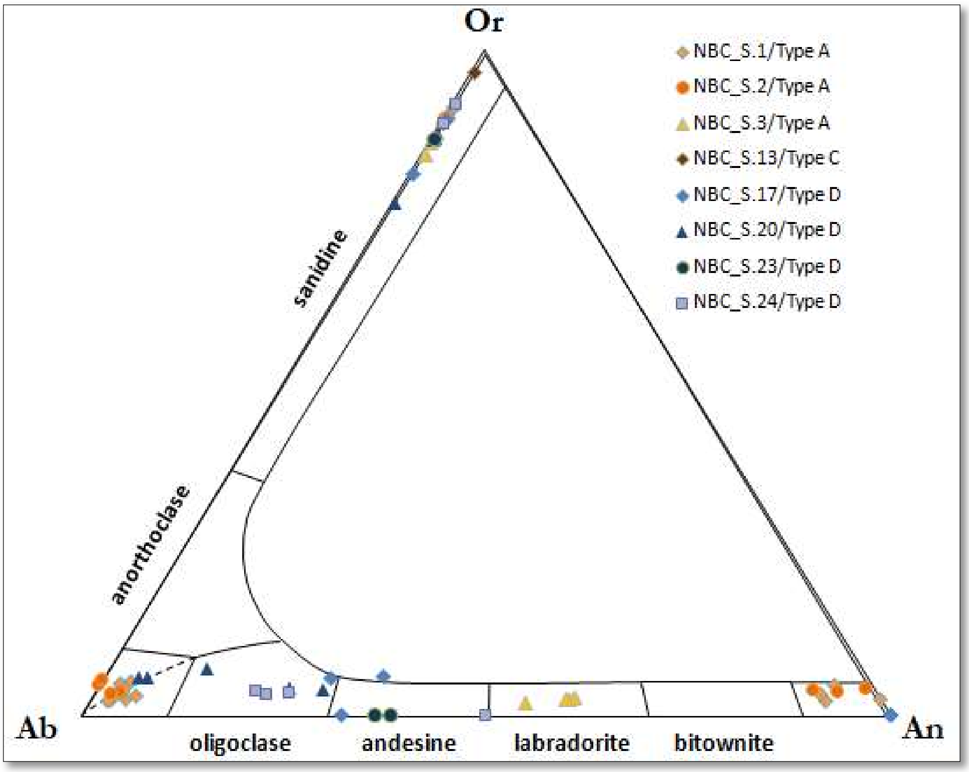

Figure S10. SEM-EDS data. Ternary diagram illustrating the composition of feldspars on selected samples from Ngongo Mbata (NBC), colour coded by sample.

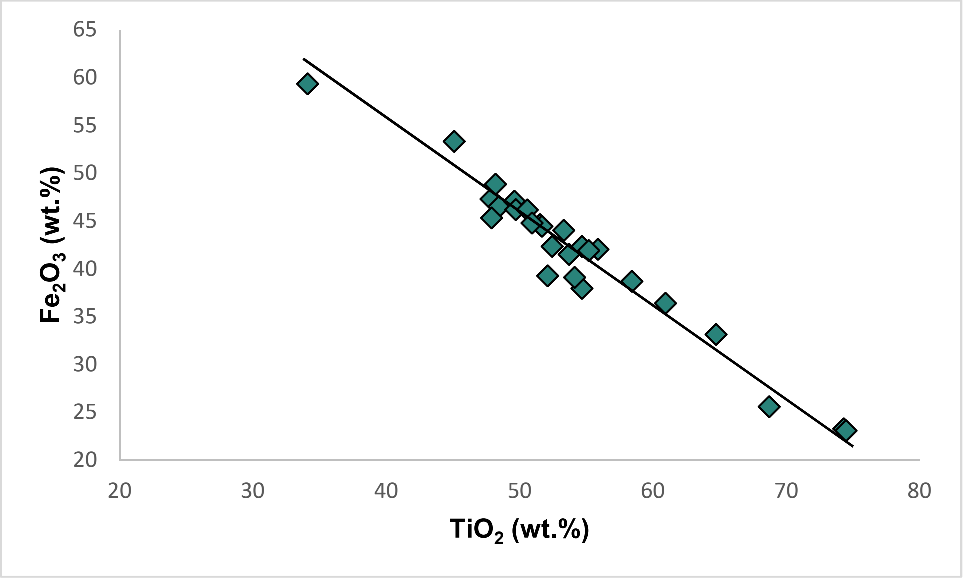

Figure S11. SEM-EDS data. Scatter plot FeO-TiO2, selected samples with identified ilmenite grains from Mbanza Kongo (MBK), Kindoki (KDK) and Ngongo Mbata (NBC).

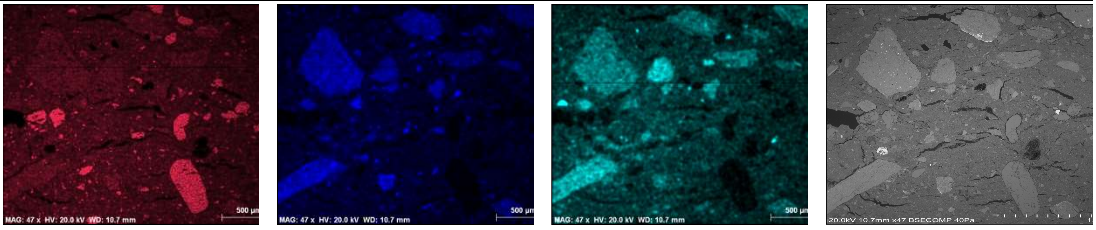

Figure S12. SEM-EDS data. Elemental distribution maps (Si, K, Mg) and backscattered electrons image of the sample KDK_S.11 of the Kindoki Group pots.

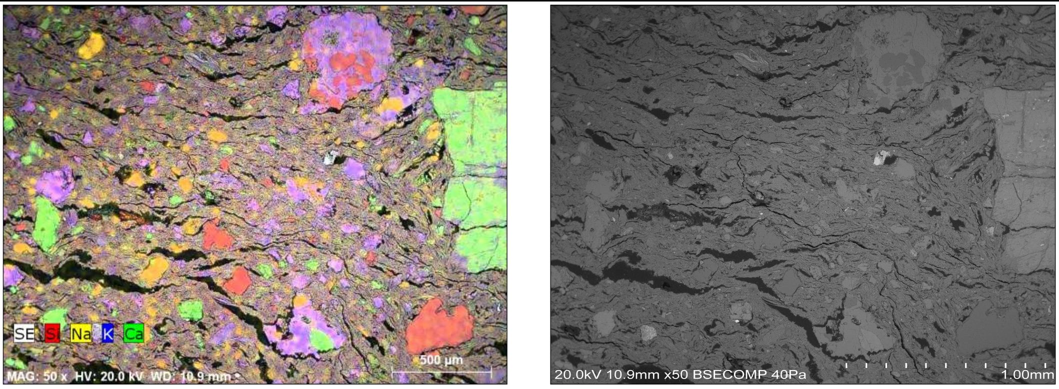

Figure S13. SEM-EDS data. Elemental distribution map and backscattered electrons image of the sample NBC_S.1 of the Type A pots.

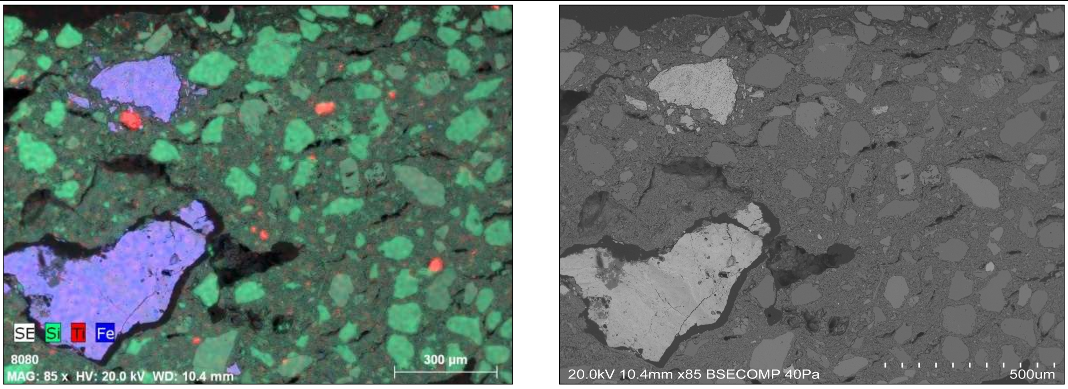

Figure S14. SEM-EDS data. Elemental distribution map and backscattered electrons image of the sample MBK_S.17 of the Type C pots.

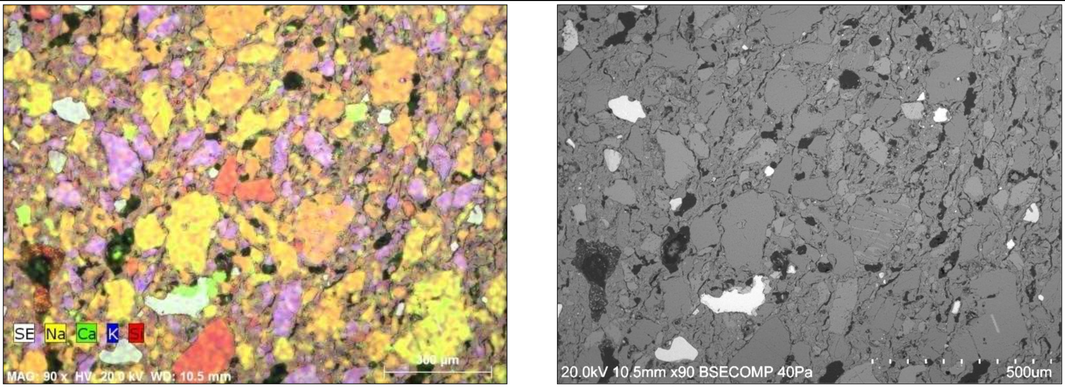

Figure S15. SEM-EDS data. Elemental distribution map and backscattered electrons image of the sample KDK_S.4 of the Type D pots.

Supplement 5. X-Ray Fluorescence spectroscopy (XRF)

Table S1. Minor elemental compositions (ppm) were obtained by the XRF analysis of the samples from Mbanza Kongo (MBK), Kindoki (KDK) and Ngongo Mbata (NBC).

Sample N°		Rb	Sr	Y	Zr	Nb	Ba	Th	Cr	Co	Ni	Cu	Zn	Ga	As	Pb	Sn	V
**MBK_S.6**	A	138	114	23	298	22	174	8	76	28	30	n.d.	91	19	14	97	30	152
**MBK_S.11**	A	170	119	18	414	23	430	15	39	23	14	n.d.	78	19	15	51	35	137
**MBK_S.9**	C	41	198	23	150	12	187	1	210	35	77	4	44	18	0	49	24	334
**MBK_S.14**	C	35	93	15	82	3	184	2	21	5	7	n.d.	16	14	4	5	29	48
**MBK_S.17**	C	76	72	36	177	26	76	15	70	20	8	15	33	20	2	25	40	139
**MBK_S.21**	C	47	84	22	246	14	226	20	20	12	6	n.d.	27	17	3	17	33	114
**MBK_S.4**	D	55	552	28	935	108	2700	1	5	20	4	n.d.	52	16	2	33	23	347
**MBK_S.15**	D	39	408	16	173	10	69	5	148	28	67	n.d.	55	17	5	18	42	218
**MBK_S.19**	D	26	205	11	252	9	n.d.	9	307	30	201	44	62	13	2	n.d.	29	219
**MBK_S.22**	D	78	443	31	645	18	882	7	14	11	7	n.d.	39	18	2	13	28	195
**MBK_S.25**	D	123	367	26	382	22	485	1	19	17	12	n.d.	49	16	1	69	35	215
**KDK_S.12**	KDK	98	30	13	166	17	599	7	53	3	43	n.d.	180	19	7	n.d.	40	192
**KDK_S.13**	KDK	72	25	29	138	15	753	10	45	7	51	n.d.	57	18	14	56	34	214
**KDK_S.15**	KDK	60	11	23	154	12	309	10	25	3	23	n.d.	44	18	4	30	27	121
**KDK_S.6**	A	154	172	21	221	17	607	9	46	17	20	n.d.	87	19	17	88	36	182
**KDK_S.8**	A	145	177	21	235	18	n.d.	n.d.	24	19	30	n.d.	83	20	39	227	31	151
**KDK_S.17**	C	42	23	53	238	18	434	11	36	9	25	n.d.	59	18	2	7	27	258
**KDK_S.19**	C	161	95	16	207	15	n.d.	5	33	15	20	n.d.	102	20	4	n.d.	28	99
**KDK_S.20**	C	49	27	16	66	12	n.d.	3	16	2	6	n.d.	29	11	5	n.d.	26	107
**KDK_S.1**	D	75	480	22	889	50	2000	14	n.d.	12	7	n.d.	33	20	1	22	40	310

Sample N°		Rb	Sr	Y	Zr	Nb	Ba	Th	Cr	Co	Ni	Cu	Zn	Ga	As	Pb	Sn	V
**KDK_S.4**	D	65	421	27	846	109	1100	0	5	9	4	n.d.	52	21	n.d.	55	20	216
**KDK_S.5**	D	71	399	22	1000	110	663	2	n.d.	10	11	n.d.	49	20	n.d.	52	40	231
**NBC_S.3**	A	16	82	7	184	9	828	8	378	57	317	n.d.	41	14	n.d.	34	23	188
**NBC_S.4**	A	14	101	21	106	22	n.d.	0	388	60	145	n.d.	35	16	n.d.	64	22	261
**NBC_S.5**	A	163	107	26	311	21	350	6	42	33	17	n.d.	100	20	18	87	42	180
**NBC_S.11**	C	38	53	25	192	16	267	8	41	9	24	n.d.	22	19	1	40	27	103
**NBC_S.12**	C	40	20	22	214	13	337	3	16	12	13	n.d.	28	19	2	31	23	97
**NBC_S.14**	C	44	44	20	287	17	252	7	20	13	12	n.d.	23	18	3	21	40	85
**NBC_S.16**	D	73	532	30	844	72	2500	9	26	26	13	n.d.	56	20	0	59	39	275
**NBC_S.17**	D	66	424	33	743	21	931	11	5	30	15	n.d.	52	21	1	17	29	180
**NBC_S.18**	D	62	579	34	697	58	2700	6	7	27	7	n.d.	59	20	1	n.d.	24	254
**NBC_S.20**	D	45	603	26	775	84	1500	7	n.d.	34	2	n.d.	45	19	1	18	40	173
**NBC_S.22**	D	118	420	24	419	17	1000	20	11	34	17	n.d.	40	17	3	9	30	182
**NBC_S.23**	D	15	69	30	135	23	56	3	282	53	121	n.d.	67	18	0	54	17	303
**NBC_S.24**	D	117	328	51	394	47	501	15	23	37	7	n.d.	50	16	1	38	37	205

***n.d.=** not detected

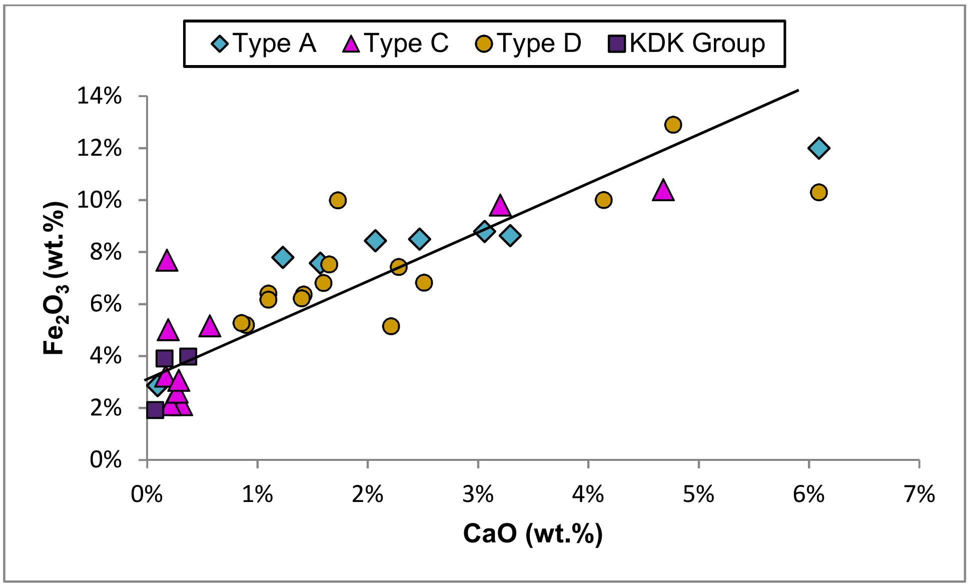

Figure S16. XRF data. Scatter plot CaO-Fe_2O_3, selected samples from Kongo kingdom pots, colour coded by typological group.

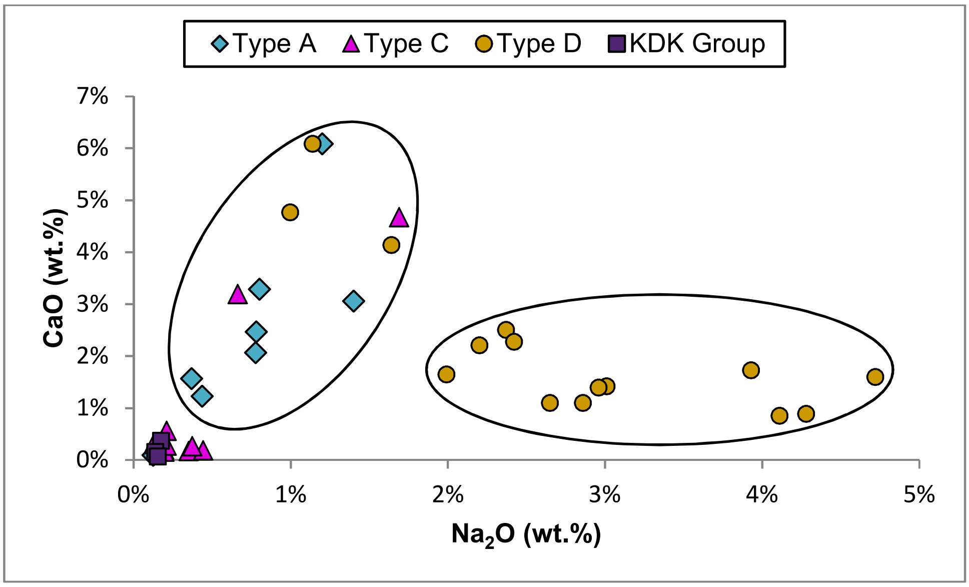

Figure S17. XRF data. Scatter plot Na_2O-CaO, selected samples from Kongo kingdom pots, colour coded by typological group.

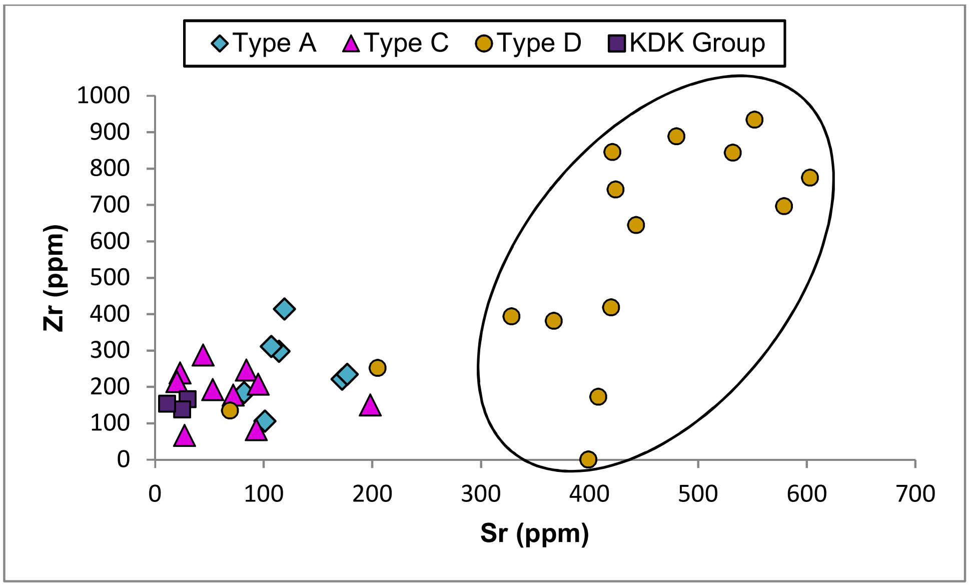

Figure S18. XRF data. Scatter plot Sr-Zr, selected samples from Kongo kingdom pots, colour coded by typological group. The sample KDK_S.5 of the Type D pots is highly concentrated in zirconium (0,10%) and is not depicted on the diagram.

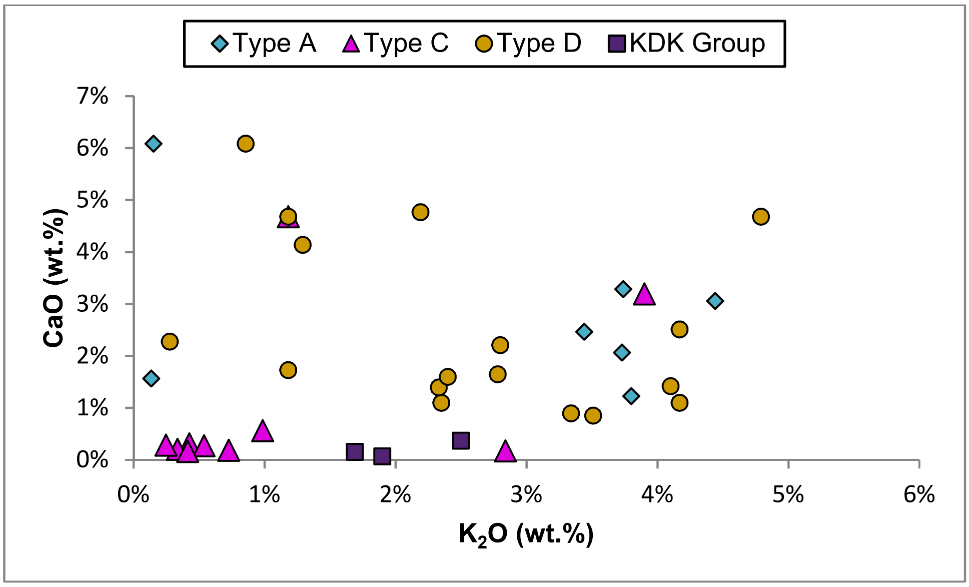

Figures S19. XRF data. Scatter plots K_2O-CaO, selected samples from Kongo kingdom pots, colour coded by typological group. No correlation indicated, as in the Rb-Sr plot for the Type A and Type D pots.

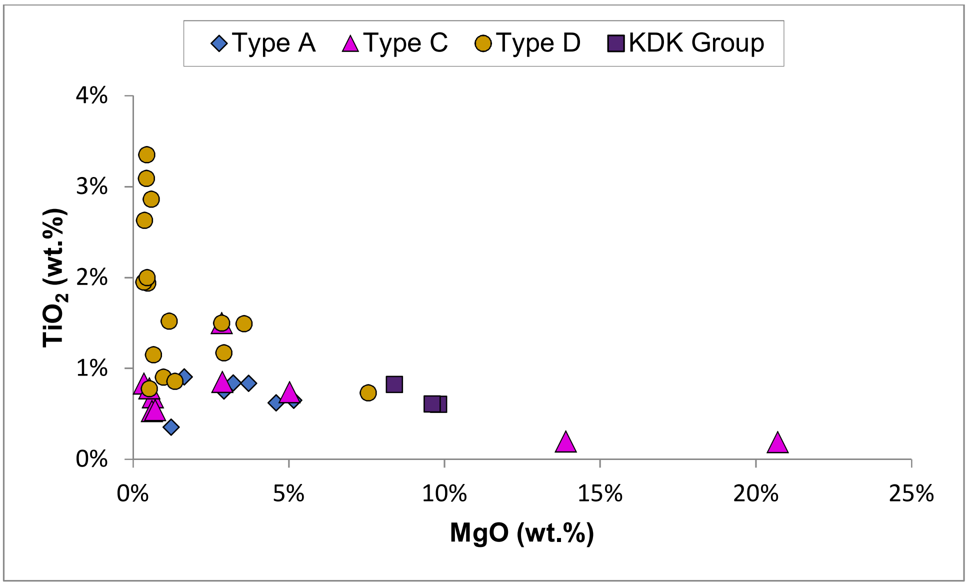

Figure S20. XRF data. Scatter plot MgO-TiO_2, selected samples from Kongo kingdom pots, colour coded by typological group.

Supplement 7. Inductively Coupled Plasma Mass Spectrometry (ICP-MS)

Table S2. Minor and trace elemental compositions (ppm) were obtained by ICP-MS analysis, of the samples from Mbanza Kongo (MBK).

Sample N°	Type	Sc	V	Co	Ni	Cu	Zn	Ga	Ge	Rb	Sr	Y	Zr	Nb	Cs	Hf	W	Pb	Bi	Th	U
**MBK_S.2**	A	16,15	143,65	14,11	40,63	38,25	131,25	24,75	3,98	134,35	245,74	43,07	78,32	26,46	4,44	2,18	1,24	43,37	0,32	17,78	4,05
**MBK_S.6**	A	15,55	122,80	22,14	25,26	30,64	124,34	24,72	3,66	142,49	104,84	25,52	90,47	23,27	6,88	2,35	1,02	92,28	0,28	18,74	4,06
**MBK_S.11**	A	14,20	113,25	18,27	19,75	34,61	106,65	22,63	2,02	170,83	111,03	19,13	200,77	30,14	6,56	5,14	1,35	87,04	0,38	17,83	5,14
**MBK_S.20**	A	14,08	104,53	21,77	27,62	34,55	127,36	25,79	1,28	201,90	108,38	26,25	96,17	22,94	6,43	2,63	0,96	113,72	0,41	17,86	3,67
**MBK_S.1**	C	30,64	273,67	13,97	54,66	105,38	80,85	30,48	3,50	70,30	40,55	39,52	229,33	34,71	3,71	5,81	1,13	25,96	0,24	12,88	3,22
**MBK_S.3**	C	10,55	103,68	5,86	29,18	34,48	65,34	15,75	1,36	29,69	19,25	31,72	81,16	9,21	2,11	2,35	0,81	17,63	0,19	9,73	7,94
**MBK_S.8**	C	16,57	162,99	38,81	30,47	37,20	25,54	17,40	1,49	14,79	79,55	10,10	85,21	9,22	0,85	2,22	n.d	10,55	0,04	6,51	2,81
**MBK_S.9**	C	32,82	234,65	34,55	104,51	68,71	64,93	22,30	2,20	41,52	200,01	22,51	112,58	15,95	2,11	3,06	0,19	7,30	0,08	5,25	1,02
**MBK_S.10**	C	26,58	208,84	12,61	55,20	87,38	92,31	25,70	3,47	71,22	63,22	39,24	209,48	33,03	5,50	4,99	0,63	15,30	0,16	10,70	2,19
**MBK_S.12**	C	8,42	86,97	4,24	16,96	22,86	54,74	13,40	1,66	35,74	16,67	72,44	86,62	9,78	1,35	2,54	0,83	14,15	0,04	9,05	7,81
**MBK_S.14**	C	5,91	40,02	1,46	7,56	12,54	21,28	11,10	2,86	29,17	72,20	16,55	42,93	4,29	2,12	1,09	0,10	11,35	0,09	4,80	1,57
**MBK_S.17**	C	17,89	126,55	2,48	17,38	79,64	44,88	23,10	1,43	76,56	62,88	32,90	144,73	29,88	2,33	3,27	0,13	10,34	0,06	6,38	1,53
**MBK_S.21**	C	7,40	61,21	2,77	7,62	13,97	28,39	15,52	0,75	33,66	58,59	16,83	76,35	15,88	1,47	2,05	0,34	11,85	0,11	9,57	1,43
**MBK_S.23**	C	14,46	104,09	28,38	50,07	37,26	69,67	14,49	0,98	50,97	166,54	10,00	96,55	9,32	1,04	2,61	n.d	16,57	0,05	7,31	0,10
**MBK_S.4**	D	10,62	80,21	7,94	3,21	12,70	72,08	21,63	1,96	55,83	526,49	20,11	56,73	125,53	0,45	1,74	0,95	12,43	0,02	6,28	1,26
**MBK_S.5**	D	11,94	94,14	24,75	3,78	8,13	75,90	23,61	2,56	50,80	534,41	23,69	60,68	80,49	0,32	1,84	0,42	12,64	0,02	6,86	1,16
**MBK_S.7**	D	16,98	116,52	14,00	25,73	26,94	58,81	23,71	2,74	63,20	154,67	21,65	106,82	24,24	2,47	3,05	0,49	20,30	0,12	19,05	3,29
**MBK_S.15**	D	21,85	169,12	37,34	57,01	60,49	77,66	22,41	1,02	38,77	397,93	21,73	44,60	12,55	1,55	1,39	4,61	28,66	0,33	11,93	2,58
**MBK_S.16**	D	17,63	144,43	27,48	42,49	37,93	73,00	20,55	1,51	54,35	133,96	13,54	100,16	13,38	1,57	2,76	0,47	14,94	0,12	17,02	4,44
**MBK_S.19**	D	46,68	168,89	66,53	267,41	132,91	95,42	15,23	1,06	26,09	210,41	16,88	38,77	11,73	0,44	1,25	n.d	11,24	0,05	3,87	0,83
**MBK_S.22**	D	10,82	74,72	9,72	8,01	17,57	52,63	19,87	1,43	76,70	399,43	28,22	48,41	22,15	0,37	1,35	n.d	13,11	0,02	8,35	0,96
**MBK_S.24**	D	10,31	79,72	7,71	3,30	7,05	74,25	21,00	1,85	56,18	557,43	23,11	63,09	84,50	0,36	1,90	0,16	10,29	0,01	6,05	1,00
**MBK_S.25**	D	15,59	133,55	30,60	22,38	58,62	67,69	22,77	1,66	127,87	341,57	26,24	50,61	23,48	4,47	1,64	0,44	20,77	0,08	18,81	4,25

***n.d.=** not detected

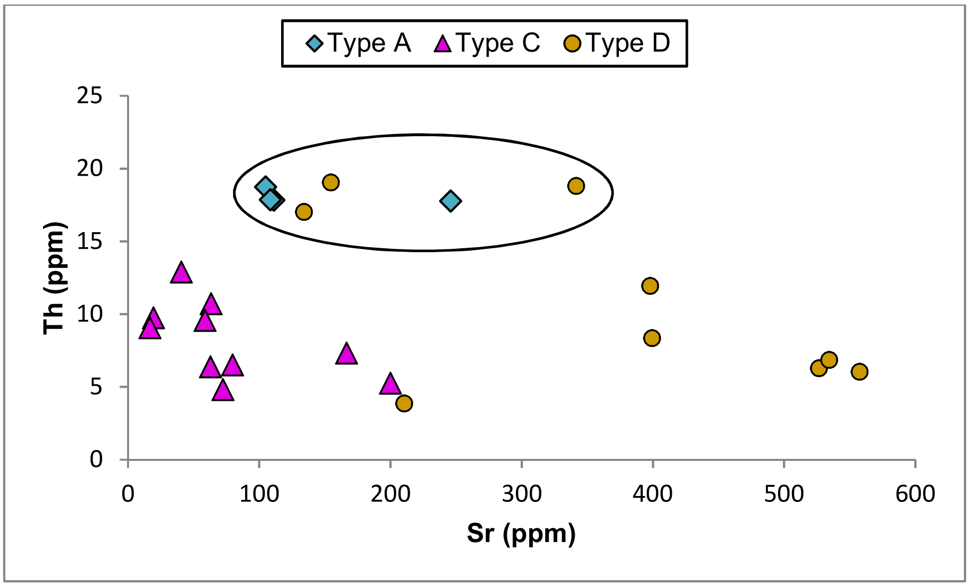

Figure S21. ICP-MS data. Scatter plot Sr-Th, selected samples from Mbanza Kongo pots, colour coded by typological group.

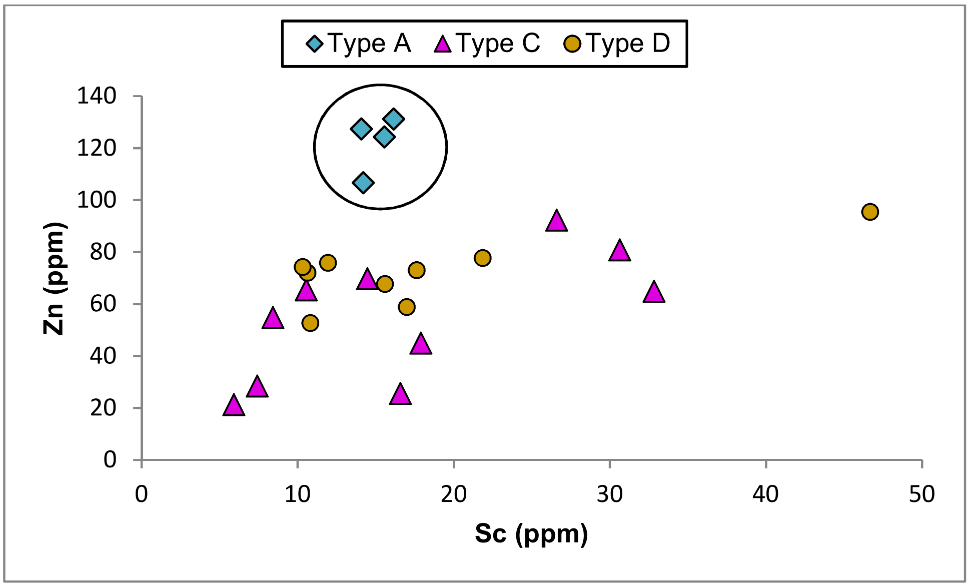

Figure S22. ICP-MS data. Scatter plot Sc-Zn, selected samples from Mbanza Kongo pots, colour coded by typological group.

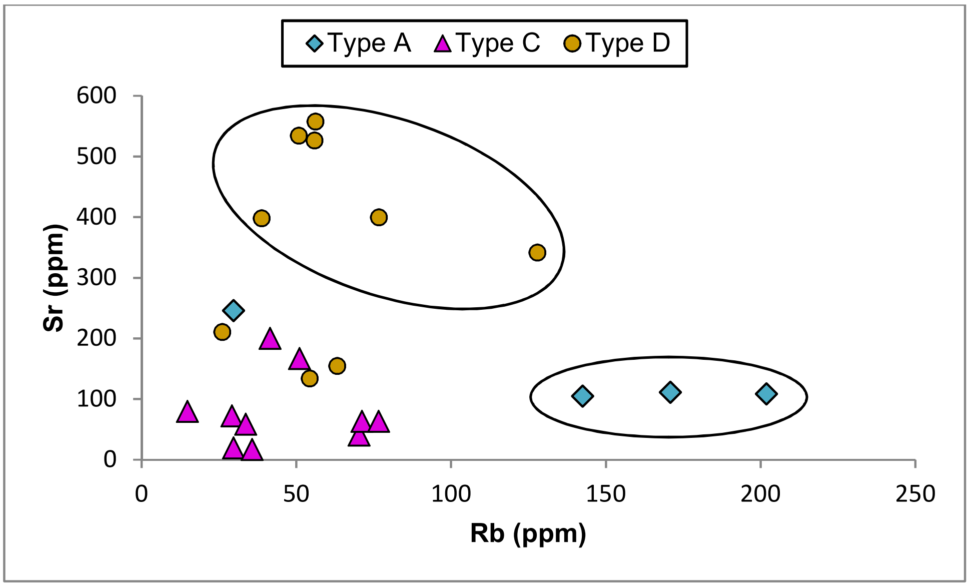

Figure S23. ICP-MS data. Scatter plot Rb-Sr, selected samples from Mbanza Kongo pots, colour coded by typological group.

Supplement 8. Sample preparation

All reagents used, during the sample preparation procedure, were of suprapur or OPTIMA grade. For cleaning, rinsing and sample dilution was used ultrapure water (18.2 MΩ.cm @ 25ºC, Milli-Q, Millipore Integral 3, Darmstadt, Germany). Nitric acid (HNO_3) (65.0%, suprapur grade, Merck) was used, diluted when required, for cleaning and sample digestion and dilution. Hydrofluoric acid (HF) (47%, OPTIMA grade, Fisher Chemicals) and hydrochloric acid (HCl) (30%, suprapur grade, Merck) were used for sample digestion. Hydrogen peroxide (H_2O_2) (30-32%, OPTIMA grade, Fisher Chemicals) was used as a chemical reagent for the organic matter destruction, on the last digestion step.

The ceramic fragments were initially sub-sampled and subdivided in two parts. The one part was used to obtain a powdered sample to be analyzed by inductively coupled plasma mass spectrometry (ICP-MS) for minor and trace element content identification, by X-Ray diffraction (XRD), either as powder for determination of the bulk mineralogical composition or in the form of oriented aggregate mounts for clay mineral identification, by thermogravimetric analysis (TGA) as powder for identification of specific mineral phases and further prepared to obtain a glass disk, required for X-Ray fluorescence spectroscopy (XRF) for major and minor elements determination. The other part was used to prepare a thin section for the petrographic analysis and variable pressure scanning electron microscopy coupled to energy dispersive X-Ray spectroscopy (VP-SEM-EDS).

ICP-MS, XRD, TGA, XRF:

The sample's surfaces were cleaned mechanically, using a multi-tool with a diamond wheel point 4,4mm to remove soil residues and eliminate any contamination. The samples were rinsed using ultrapure water and dried at 40 °C for 24 h. Afterwards, the samples were ground up with an agate mortar and pestle to a fine homogeneous powder approximately 60µm grain size. Ultrapure water and HNO_3 (at 6.5%) were used to clean the diamond wheel point and the mortar/pestle between the samples to avoid any contamination by traces.

Prior to ICP-MS analysis, the powdered samples (≅100mg) were first digested by acid attack on a hotplate. In perfluoroalkoxy (PFA) closed beakers, the selected samples were digested with a mixture of 0,5mL of concentrated HNO_3 (65%) and 2 mL of HF (47%), for 48 hours at 140-150°C for silicates digestion. Following cooling, the samples were dried (not completely, to avoid fluorides precipitations and stabilisation) by evaporation on a hotplate. Then, the samples were re-suspended in 2mL of freshly prepared aqua-regia solution [HNO_3 and HCl (1:3)] to digest under reflux for 24 hours on a hotplate at 120-140°C. Following cooling, the samples were dried once again on a hotplate. The last digestion step included 2 mL of HNO_3 (65%) for 24 hours on a hot plate at 120-140°C with closed beakers. Due to the presence of organic matter, H_2O_2 (30-32%) was added and the samples were allowed to complete the digestion. Following drying, the samples were finally re-suspended with 1.6 mL of 65% HNO_3 and 3 mL of ultrapure water and after transferred to PFA volumetric flasks and fulfilled with ultrapure water up to 50 mL, to reach a solution matrix of 2% HNO_3. The samples were diluted 100 fold for minor elements with 2% HNO_3 at a final volume of 10 mL. For trace elements determination the samples were analysed with no dilution.

The powdered samples were analyzed by XRD for the determination of the bulk mineralogical composition. The ones displaying peaks in clay-minerals were analyzed

as oriented aggregate mounts, after being prepared as follows. The powdered samples were mixed with ultrapure water and after settling for fifteen seconds, the material was extracted by using a pipette and applied on a glass substrate, enabling the clay minerals to lie flat. This procedure was performed in duplicates. Then the oriented aggregate mounts were analyzed dried in room temperature. The samples were subjected into standard treatments, (1) glycolation with ethylene glycol in the oven in 60°C for 12 hours and (2) heating to 400°C and to 550°C, placing the samples in the oven at increasing temperature for fifteen minutes and at 400°C and 550°C, respectively, for thirty minutes. XRD analysis was performed on the non- treated samples and after each treatment.

TGA was performed using a 25-35 mg powder of each of the selected samples.

The powdered samples were further prepared for XRF analysis. A 1,2g of each powdered sample was mixed with 12 g of fusion flux (Li-tetraborate) (Lithium Tetraborate 49.75% ($Li_2B_4O_7$)/ Lithium Metaborate 49.75% ($LiBO_2$)/ Lithium Iodide 0.5%). The powder mixture was fused in 1065°C and then cast into a glass disk by using a fusion instrument.

Petrographic analysis, VP-SEM-EDS:

Thin sections were prepared for petrographic and VP-SEM-EDS analysis. For the preparation, the samples were cleaned with a plastic brush to remove the soil residues and washed using ultrapure water. They were allowed to dry at 40 °C for 24 h. Then, they were embedded in epoxy resin (25:3) (Epofix Fix, Struers A/S; 24 h. hardening time). The cross-sections were then polished using Silicon Carbide papers of different grain sizes (Struers, FEPA P # 320, 500, 800, 1200, 2000 and 4000, where 4000 is 5μm). Afterwards, the cross-sections were glued on a glass with epoxy resin and cut with the saw to a distance of around 1.70cm from the glass. The thin-sections were ground smooth by the saw and then, they were polished using a rotation disk and Silicon Carbide powder (P # 400, 1000), in order the sample to reach the required thickness of around 30μm.

Supplement 9. Inductively Coupled Plasma - Mass Spectrometry (ICP-MS), operational conditions, analytes of interest, LoD and LoQ

Table S3. Operational conditions used for the minor and trace elemental analysis by ICP-MS.

**Acquisition Mode**	**Spectrum**
**Spectrum Mode Option**	Q2 Peak Pattern: 1 Point<br>Replicates: 3<br>Sweeps/Replicate: 10
**Scan Type**	MS/MS
**Plasma Parameters**	
**RF Power**	1550W
**RF Matching**	1.8V
**Sample Depth**	10mm
**Carrier Gas (Ar)**	1.01 $L.min^{-1}$
**Plasma Gas (Ar)**	15 $L.min^{-1}$
**Nebulizer Pump**	0.10 rps
**Collision Cell (CRC)**	
**Collision Gas: He**	Flow: 4.0 $mL.min^{-1}$
**Reaction Gas: O_2**	Flow: 0.5 $mL.min^{-1}$
**Reaction Gas: NH_3**	Flow: 1.5 $mL.min^{-1}$(in 1mL.min-1 of He)
**Analysis Mode**	No gas, He, O_2, NH_3
**Dwell time**	
**0.3s**	^{45}Sc, ^{51}V, ^{59}Co, ^{60}Ni, ^{63}Cu, ^{66}Zn, ^{71}Ga,^{72}Ge, ^{85}Rb, ^{88}Sr, ^{89}Y, ^{90}Zr, ^{93}Nb
**0.5s**	^{133}Cs, ^{137}Ba, ^{139}La, ^{140}Ce, ^{141}Pr, ^{146}Nd, ^{147}Sm, ^{153}Eu, ^{157}Gd, ^{159}Tb, ^{163}Dy, ^{165}Ho, ^{166}Er, ^{169}Tm, ^{172}Yb, ^{175}Lu, ^{178}Hf, ^{181}Ta, ^{182}W, ^{208}Pb, ^{209}Bi, ^{232}Th, ^{238}U

Table S4. Selected isotopes, collision/reaction (CRC) gas mode along with the LoD and LoQ.

Isotopes	CRC Gas Mode	LoD (ppb)	LoQ (ppb)	Isotopes	CRC Gas Mode	LoD (ppb)	LoQ (ppb)
^{45}**Sc**	He	0,448	4,478	^{146}**Nd**	no-gas	0,050	0,499
^{51}**V**	He	0,055	0,547	^{147}**Sm**	no-gas	0,056	0,561
^{59}**Co**	He	0,041	0,413	^{153}**Eu**	no-gas	0,061	0,614
^{60}**Ni**	He	1,096	10,957	^{157}**Gd**	no-gas	0,068	0,683
^{63}**Cu**	He	0,046	0,463	^{159}**Tb**	no-gas	0,064	0,638
^{66}**Zn**	He	0,212	2,124	^{163}**Dy**	no-gas	0,081	0,808
^{71}**Ga**	He	0,243	2,434	^{165}**Ho**	no-gas	0,081	0,810
^{72}**Ge**	He	0,181	1,810	^{166}**Er**	no-gas	0,090	0,904
^{85}**Rb**	He	0,207	2,072	^{169}**Tm**	no-gas	0,102	1,021
^{88}**Sr**	He	0,093	0,926	^{172}**Yb**	no-gas	0,121	1,206
^{89}**Y**	He	0,018	0,180	^{175}**Lu**	no-gas	0,126	1,264
^{90}**Zr**	He	0,046	0,460	^{181}**Ta**	no-gas	0,008	0,078
^{93}**Nb**	He	0,061	0,611	^{178}**Hf**	no-gas	0,051	0,505
^{133}**Cs**	no-gas	0,051	0,506	^{182}**W**	no-gas	0,814	8,137
^{137}**Ba**	no-gas	0,028	0,276	^{208}**Pb**	no-gas	0,102	1,021
^{139}**La**	no-gas	0,032	0,324	^{209}**Bi**	no-gas	0,367	3,675
^{140}**Ce**	no-gas	0,042	0,418	^{232}**Th**	no-gas	0,123	1,232
^{141}**Pr**	no-gas	0,045	0,453	^{238}**U**	no-gas	0,110	1,097

***n.d.**= not determined

Supplement 10. Analytical methods applied on the studied material

Table S5. The samples discussed in this study by the analytical method applied.

Type	Sample N	XRD	TGA	petrography	VP-SEM-EDS	XRF	ICP-MS
A	MBK_S.2	X		X			X
	MBK_S.6	X		X	X	X	X
	MBK_S.11	X		X		X	X
	MBK_S.20	X		X			X
C	MBK_S.1	X			X		X
	MBK_S.3	X		X			X
	MBK_S.8	X					X
	MBK_S.9	X				X	X
	MBK_S.10	X					X
	MBK_S.12	X		X			X
	MBK_S.14	X	X	X		X	X
	MBK_S.17	X		X	X	X	X
	MBK_S.21	X		X		X	X
	MBK_S.23	X		X			X
D	MBK_S.4	X		X	X	X	X
	MBK_S.5	X		X	X		X
	MBK_S.7	X		X			X
	MBK_S.15	X		X	X	X	X
	MBK_S.16	X		X			X
	MBK_S.19	X		X		X	X
	MBK_S.22	X		X		X	X
	MBK_S.24	X		X	X		X
	MBK_S.25	X		X		X	X
KDK	KDK_S.11	X		X	X		
	KDK_S.12	X		X		X	
	KDK_S.13	X	X	X	X	X	
	KDK_S.14	X		X			
	KDK_S.15	X				X	
	KDK_S.16	X		X			
A	KDK_S.6	X		X	X	X	
	KDK_S.7	X		X	X		
	KDK_S.8	X		X		X	
	KDK_S.9	X		X	X		
C	KDK_S.17	X		X		X	
	KDK_S.18	X		X			
	KDK_S.19	X		X		X	
	KDK_S.20	X	X	X		X	
	KDK_S.21	X		X			
	KDK_S.22	X		X			
	KDK_S.23	X		X			
	KDK_S.25	X		X			
D	KDK_S.1	X		X		X	
	KDK_S.2	X		X			
	KDK_S.3	X		X	X		
	KDK_S.4	X		X	X	X	
	KDK_S.5	X		X	X	X	
A	NBC_S.1	X		X	X		
	NBC_S.2	X		X	X		
	NBC_S.3	X		X	X	X	
	NBC_S.4	X		X		X	

	**NBC_S.5**	X		X		X	
**C**	**NBC_S.9**	X		X			
	**NBC_S.10**	X					
	**NBC_S.11**	X		X		X	
	**NBC_S.12**	X		X		X	
	**NBC_S.13**	X		X	X		
	**NBC_S.14**	X		X	X	X	
**D**	**NBC_S.16**	X		X		X	
	**NBC_S.17**	X		X	X	X	
	**NBC_S.18**	X		X		X	
	**NBC_S.19**	X		X			
	**NBC_S.20**	X		X	X	X	
	**NBC_S.21**	X		X			
	**NBC_S.22**	X		X		X	
	**NBC_S.23**	X		X	X	X	
	**NBC_S.24**	X		X	X	X	
	**NBC_S.25**	X		X			
	**TOTAL N**	67	3	61	24	35	23

Supplement 11. Shapefile of the geological map created in ArcGIS Pro 2.9.1 software

Attached as a separate file, can be downloaded by Supplementary Information 2.

ADDITIONAL REFERENCES

66. ICOMOS, Mbanza Kongo, vestiges of the capital of the former Kingdom of Kongo (Angola), No 1511, *ICOMOS report* (2017).

67. Costa, M., *et al.* Determining the provenance of the European glass beads of Lumbu (Mbanza Kongo, Angola). *Microchemical Journal*, **154,** https://doi.org/10.1016/j.microc.2019.104531 (2020).
